# Digital Phenotyping and Patient-Generated Health Data for Outcome Measurement in Surgical Care: A Scoping Review

**DOI:** 10.3390/jpm10040282

**Published:** 2020-12-15

**Authors:** Prakash Jayakumar, Eugenia Lin, Vincent Galea, Abraham J. Mathew, Nikhil Panda, Imelda Vetter, Alex B. Haynes

**Affiliations:** 1 Department of Surgery and Perioperative Care, Dell Medical School, The University of Texas at Austin, Austin, TX 78712, USA; eugenia.lin@austin.utexas.edu (E.L.); joshuamathew41@gmail.com (A.J.M.); alex.haynes@austin.utexas.edu (A.B.H.); 2 School of Medicine, New York Medical College, Valhalla, NY 10595, USA; vgalea@student.nymc.edu; 3 Department of Surgery, Massachusetts General Hospital, Harvard Medical School, Boston, MA 02114, USA; Nikhil.panda@mgh.harvard.edu; 4 Department of Medical Education, Dell Medical School, The University of Texas at Austin, Austin, TX 78712, USA; imelda.vetter@austin.utexas.edu

**Keywords:** digital phenotyping, patient-generated health data, patient monitoring, activity tracking, wearables, sensors, patient-reported outcome measures, surgery

## Abstract

Digital phenotyping—the moment-by-moment quantification of human phenotypes in situ using data related to activity, behavior, and communications, from personal digital devices, such as smart phones and wearables—has been gaining interest. Personalized health information captured within free-living settings using such technologies may better enable the application of patient-generated health data (PGHD) to provide patient-centered care. The primary objective of this scoping review is to characterize the application of digital phenotyping and digitally captured active and passive PGHD for outcome measurement in surgical care. Secondarily, we synthesize the body of evidence to define specific areas for further work. We performed a systematic search of four bibliographic databases using terms related to “digital phenotyping and PGHD,” “outcome measurement,” and “surgical care” with no date limits. We registered the study (Open Science Framework), followed strict inclusion/exclusion criteria, performed screening, extraction, and synthesis of results in line with the PRISMA Extension for Scoping Reviews. A total of 224 studies were included. Published studies have accelerated in the last 5 years, originating in 29 countries (mostly from the USA, *n* = 74, 33%), featuring original prospective work (*n* = 149, 66%). Studies spanned 14 specialties, most commonly orthopedic surgery (*n* = 129, 58%), and had a postoperative focus (*n* = 210, 94%). Most of the work involved research-grade wearables (*n* = 130, 58%), prioritizing the capture of activity (*n* = 165, 74%) and biometric data (*n* = 100, 45%), with a view to providing a tracking/monitoring function (*n* = 115, 51%) for the management of surgical patients. Opportunities exist for further work across surgical specialties involving smartphones, communications data, comparison with patient-reported outcome measures (PROMs), applications focusing on prediction of outcomes, monitoring, risk profiling, shared decision making, and surgical optimization. The rapidly evolving state of the art in digital phenotyping and capture of PGHD offers exciting prospects for outcome measurement in surgical care pending further work and consideration related to clinical care, technology, and implementation.

## 1. Introduction

Technology-enabled solutions that capture patient-generated health data (PGHD)—data related to activity, mobility, cognition, behavior, mood and social interactions—are rapidly evolving with the aim of a more personalized, patient-centered, and data-driven approach to the delivery of surgical care [1,2,3,4,5]. The concept of “digital phenotyping” was first coined in 2015 by J.P Onnela as the moment-by-moment quantification of individual human phenotypes in situ using data related to activity, behavior, and communications from personal digital devices, such as smartphones and wearable sensors (wearables) [6,7,8,9,10]. While the first smartphones were developed around 1992, wider utilization and applications capturing PGHD occurred toward the late 2000s. The acquisition of PGHD in the form of patient-reported outcome measurements (PROMs) is commonplace in clinical research and increasingly common in clinical care. PROMs are questionnaires that quantify the patient’s perspective of their physical, emotional, and social health, and are commonly collected using tablet devices and web-based, online portals [11,12,13,14,15]. The electronic capture and utility of PROMs has transformed the evaluation of health outcomes in surgical research, partly due to well-defined surgical pathways and time points during the preoperative baseline to postoperative recovery and rehabilitation [12,16]. However, the adoption of PROMs in clinical practice is limited by the burden placed on patients to interpret and complete surveys, is often restricted to the clinical encounter, and associated with several administrative and logistical barriers in sustaining longitudinal data collection, especially in busy, resource-limited settings [15,17].

### 1.1. Rationale

The continuous capture of passive PGHD in “real time” may overcome these limitations via digital phenotyping. However, little is known around digital phenotyping and PGHD in the context of outcome measurement in surgical care. An individual’s digital phenotype and how they interact with these devices aims to provide dynamic insights around the impact of a given condition on the patient’s lived experience, both within and outside health care settings. This rich data source may augment the way we traditionally acquire health information via physical assessment (clinical history and examination), and investigations (vital signs monitoring, laboratory tests, medical imaging), and further advance the tracking and surveillance of health, enhance decision making at the point of care, trigger the timely detection of clinical deterioration, and better predict surgical outcomes [13,14,18]. While a growing evidence base supports the value of digital phenotyping and PGHD to provide actionable data and targeted interventions, few have comprehensively characterized this technology in surgery or mapped current concepts for driving research and development in this field. The overarching goal of this study was to conduct a rapid scoping review of digital phenotyping and PGHD for outcome measurement in surgery to generate a repository of evidence for the current state of the art, identify knowledge gaps, and guide recommendations for future work.

### 1.2. Objectives

The primary objective was to map the application of digital phenotyping and digitally captured active and passive PGHD for outcome measurement in surgical care by study characteristics, clinical characteristics, technological/data characteristics, and functional characteristics. The secondary objective was to synthesize the body of evidence to define specific areas of further work necessary to translate this technology from research bench to surgical practice. Ultimately, this review aims to inform stakeholders in advancing the field of patient-centered digital health and outcome measurement in surgical care.

## 2. Materials and Methods

### 2.1. Study Design

We performed a rapid scoping review as a streamlined approach to synthesizing evidence for emergent research and development in this field [19,20,21]. We started with a strategic search applied to multiple electronic databases using search terms related to key concepts within our primary and secondary objectives. This was followed by a stepwise process of screening, data extraction, and synthesis.

### 2.2. Protocol and Registration

The protocol was developed a priori, guided by the Preferred Reporting Items for Systematic Reviews and Meta-analysis—Extension for Scoping Reviews (PRISMA-ScR) (Appendix A) [20], and study registered prospectively with the Open Science Framework, Center for Open Science (Registration No. url: osf.io/p9c7u).

### 2.3. Eligibility Criteria

Eligibility criteria were as follows: studies focused on adult patients undergoing any form of surgical care at any phase along the care pathway (i.e., preoperative evaluation, perioperative care, postoperative recovery and rehabilitation), involving personal digital devices used to capture active and/or passive PGHD, describing outcome measurement(s) across any health domain, within original studies (prospective, retrospective, technical feasibility) in peer-reviewed journals that were available in the English language. Studies were excluded if they involved pediatric and adolescent patients, non-surgical contexts, lacked capture of any form of PGHD, involved digital solutions to collect and synthesize PROMs only, or were reviews, commentaries, case studies, without original data, and not available in the English language.

### 2.4. Search and Data Sources

We developed a search strategy guided by our lead institutional librarian [IV], who is experienced in performing systematic reviews. Following rounds of refinement among the research team we defined and combined terms related to “digital phenotyping and PGHD” (concept A), “outcome measurement” (concept B), and “surgical care” (concept C) (Appendix B). Search engines were selected by consensus among authors and our librarian expert then deployed the final search strategy across the following electronic bibliographic databases: PubMed (NLM), Web of Science (Clarivate Analytics, Philadelphia, PA, USA), Cochrane Library (Wiley, Hoboken, NJ, USA), and IEEE Xplore; Databases were searched on 1 June 2020 and refreshed on 1 July of 2020 to ensure we acquired an up-to-date set of articles before reporting findings. No limits were set in publication dates for search purposes, however results spanned years from 1994 to 2020. Search results were limited by language (English only) and resource type (journal articles only). Search results were exported into and deduplicated with the citation management tool EndNote. The search was supplemented by scanning reference lists of relevant reviews.

### 2.5. Data Screening

Three investigators (EL/VG/JM) independently screened titles and abstracts from the full set of articles based on eligibility criteria. For quality control and to increase consistency among reviewers, all reviewers initially screened a set of 25 publications at the outset and discussed the results before continuing with the screening process. Subsets of articles from batches were cross-checked by investigators (PJ/EL/VG) for consistency and quality assurance. Excluded studies were coded with reasons for exclusion using the criteria established a priori. Any differences in judgment on inclusion/exclusion of studies were resolved by group discussions with the senior investigator (AH) as needed. Full-text articles were retrieved for further independent review and final assessment for eligibility (PJ/EU/VG). Number of articles screened, and articles excluded including duplicates were logged for each source of evidence and presented in a PRISMA flow diagram (Appendix C). The final study set for data extraction was thus identified.

### 2.6. Data Charting Process

Two investigators (PJ/EL) jointly developed the data charting system including electronic forms for screening, data extraction, and synthesis of relevant information (Microsoft Excel, v16.21, USA). The screening form logged articles for inclusion/exclusion, allowed tagging of queried citations for further discussion and recording reasons for excluding articles. The extraction form included parameters developed in relation to our primary objective, i.e., study characteristics, clinical characteristics, technological/data characteristics and functional characteristics. Data items for each category were selected by four investigators (PJ/EL/VG/JM) who charted data independently. These investigators regrouped at regular points throughout the screening and extraction phase to discuss and iterate the data charting parameters. Any inconsistencies were resolved by additional input from the senior author (AH) as needed.

### 2.7. Data Items and Extraction

We finally abstracted data from the full text (PDFs) of the final set of selected articles on: study characteristics (lead author, study year, country of origin, study design, total number of patients), clinical characteristics (surgical specialty, surgical procedure, point of application along care pathway), technological and data characteristics (type of device including brand/proprietary names, type of data), and functional characteristics (types of clinical function and utility) with additional notes to document salient points.

### 2.8. Appraisal of Individual Sources of Evidence

We focused on presenting the results as a “map” of data utilizing data visualizations and data tabulations along with a descriptive narrative as per published guidelines, in keeping with a broad and scoping systematic review [20,22]. While we closely reviewed the full text articles during the data extraction phase, we did not proceed with a formal critical appraisal partly given the heterogeneity of the study set (varying study designs in particular), and partly due to the lack of a universal and validated quality assessment tool.

### 2.9. Synthesis of Results

We synthesized results using coding and grouping of relevant data elements using our electronic database. with descriptive analysis using frequencies and percentages within each category of extracted data. Following consensus discussions on metrics of interest by three investigators [PJ/EL/VG], we proceeded to tabulate data and generate visualizations using a data analytics package (Tableau, 2020. v3.0, Mountain View, CA, USA). Visualizations included a geographical chart of country of origin for selected articles; bubble charts and other standard charts for other metrics of relevance, and a Sankey-type flow diagram (@SankeyMATIC, Virginia, USA) to provide an overview of the specific inter-relation between technological, data and functional characteristics.

## 3. Results

### 3.1. Initial Evaluation and Selection of Studies

**Figure 1:**
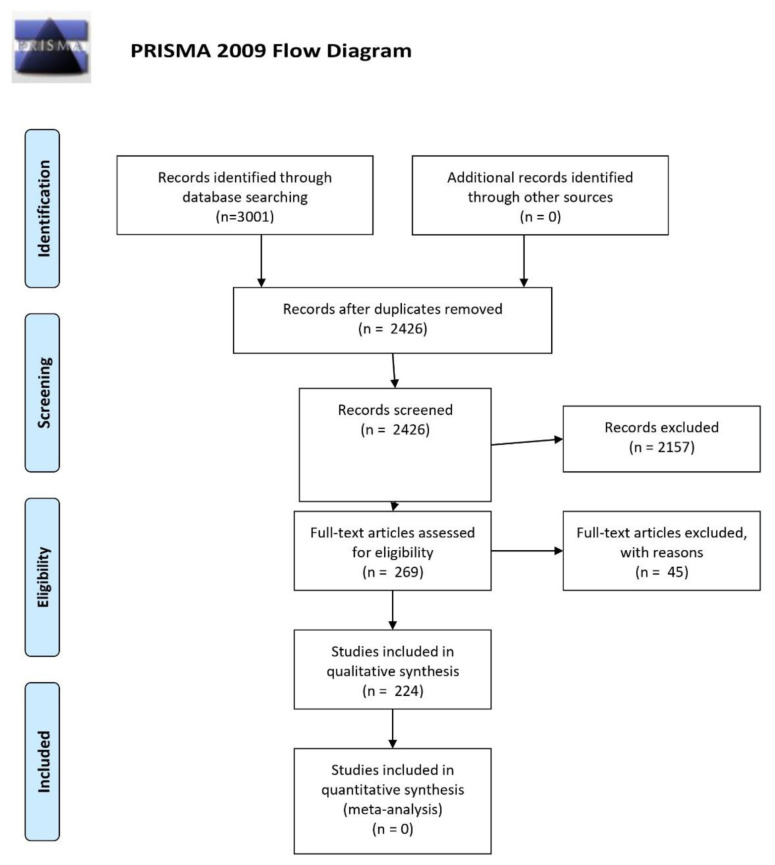
PRISMA Flow Diagram of study identification, screening, eligibility, and inclusion in final review [23].

A total of 3001 citations were generated from the original literature search and after adjusting for duplicates (*n* = 575), 2426 remained for screening. After reviewing titles and abstracts, 2157 were excluded by criteria leaving 269 publications for full-text review. A further 45 studies were excluded based on a lack of alignment with our study objectives and leaving a final set of 224 articles (Table 1) (Figure 1) (Appendix B).

### 3.2. Study Characteristics

**Figure 2:**
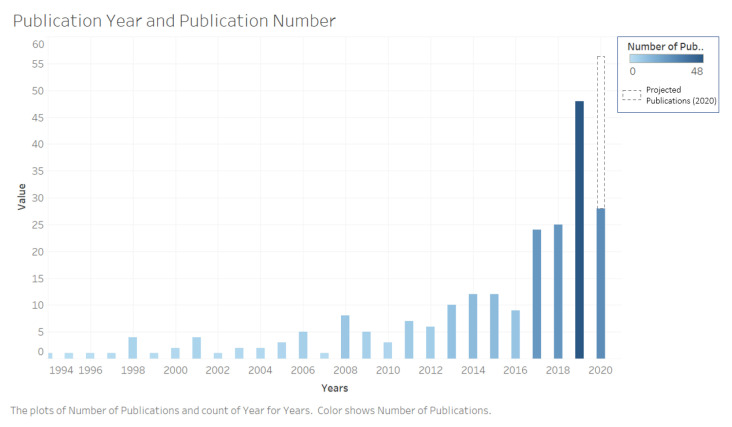
Total number of publications by year for studies related to digital phenotyping and patient-generated health data for outcome measurement in surgical care.

**Figure 3:**
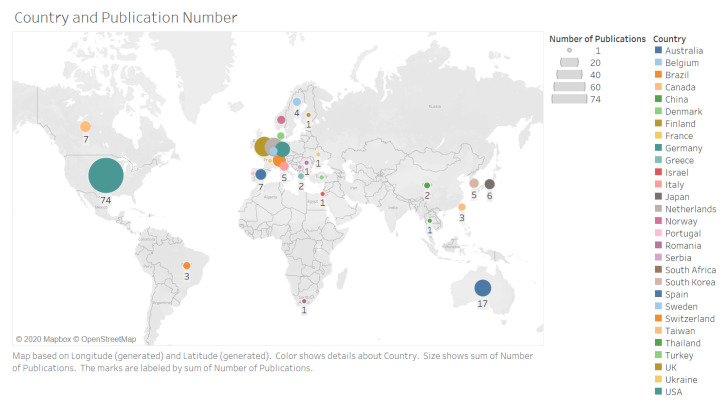
Geographical distribution of studies by country of origin where work was conducted. Studies originated from a total of 29 different countries.

**Figure 4:**
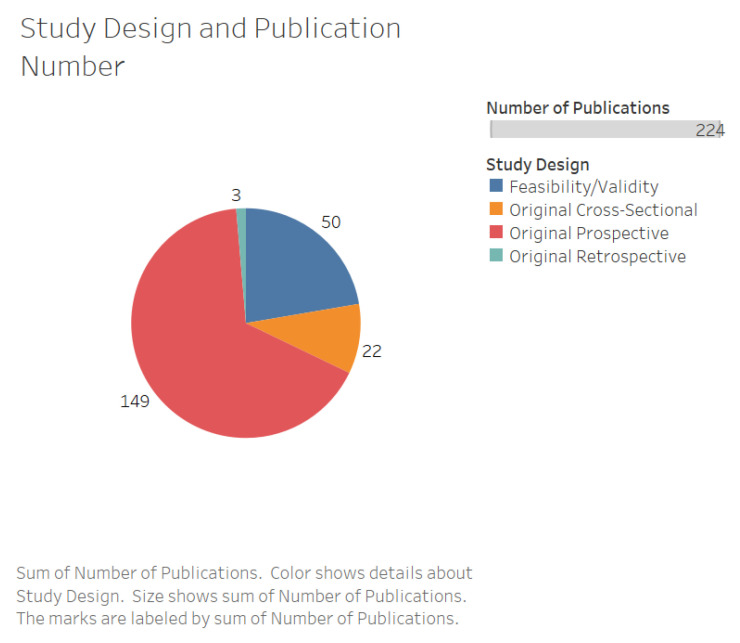
Pie chart representing the number of studies by study design.

The number of studies increased over time (Figure 2). Studies originated from 29 countries with the majority performed in the USA (*n* = 74, 33%) (Figure 3). The majority of studies featured original prospective work (*n* = 149, 67%), and a substantial proportion of studies involved technical validation and feasibility of digital solutions (*n* = 50, 22%) (Figure 4). The cohorts of patients involved in these studies ranged from 5 to 406 participants.

### 3.3. Clinical Characteristics

**Figure 5:**
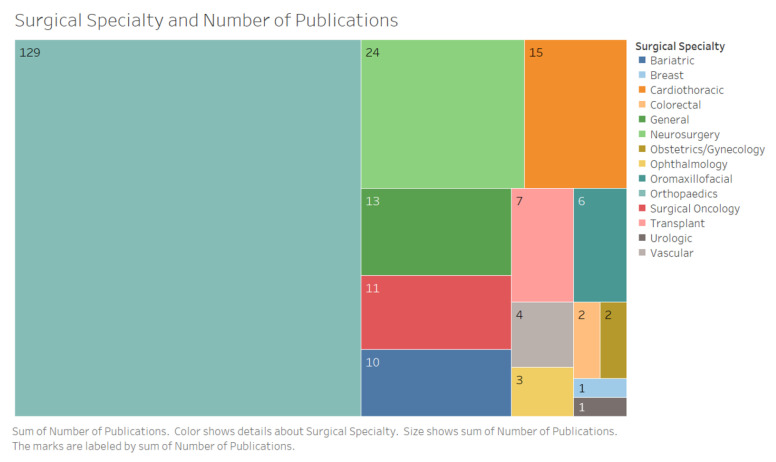
Area chart representing number of studies by surgical specialty. Studies spanned a total of 14 surgical specialties.

Studies spanned 14 surgical specialties with the majority being performed in the context of orthopedic surgery (*n* = 129, 58%) and procedures including total joint replacement, fracture and soft tissue trauma reconstruction, joint fusion, brachial plexus injury, rotator cuff repair, anterior cruciate ligament reconstruction and carpal tunnel release (Figure 5) (Appendix D). The majority of studies were conducted in the postoperative phase (*n* = 210, 94%).

### 3.4. Technological/Data Characteristics

**Figure 6:**
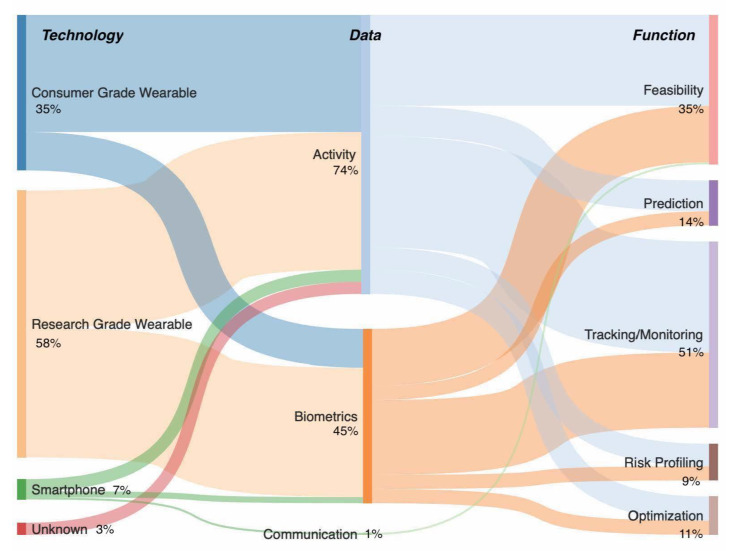
A Sankey-type flow diagram representing flow of studies by technology, data and function.

Overall, the majority of studies involved research-grade wearables (i.e., non-commercially available wearable sensors/sensors for research purposes only) (*n* = 129, 58%), and consumer-grade wearables (i.e., commercially available wearable sensors produced for the consumer market but used for scientific evaluation) (*n* = 78, 35%) over smartphone (*n* = 15, 7%) or other devices (*n* = 6, 3%). There was a predominant focus on capturing activity (*n* = 165, 74%), and biometric data (*n* = 100, 45%), as opposed to communications data (*n* = 2, 1%) (Figure 6). As a single publication could fall under multiple technological or data characteristic categories, the summed percentages are greater than 100%.

The width of each flow is proportional to number of studies channeled from one category to another, i.e., the flow of number of articles published by technology type that involved the capture of activity, biometric and/or communications data in order to provide a given function

### 3.5. Functional Characteristics

**Figure 7:**
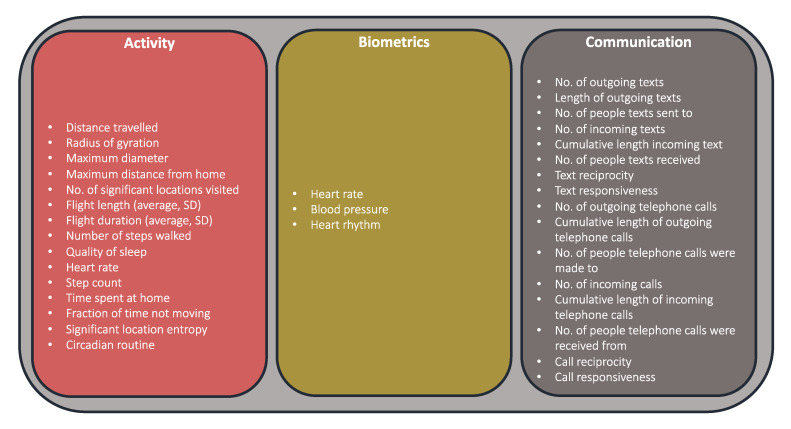
The “Activity-Biometrics-Communication” Framework of Activity, Biometric, and Communications data points captured using Personal Digital Devices. This does not include capture of patient-reported outcome measurements (PROMs) and other survey questions via Smartphone applications/other devices.

The focus of the majority of studies was on tracking/monitoring of surgical patients (*n* = 115, 51%), and assessment of technical feasibility (*n* = 78, 35%), versus prediction of surgical outcomes (*n* = 32, 14%), risk profiling (*n* = 20, 9%), and surgical optimization (*n* = 25, 11%). A wide range of technologies were utilized such as activity trackers, smartphone applications, research- and commercial-grade wearables, and other sensors (Appendix E) alongside numerous types of activity, biometric, and communication-related data points (Figure 7). As a single publication could be categorized in multiple functional characteristics, the sum of the values and sum of percentages is higher than 224 and 100%, respectively.

Notably, various patient-reported outcome measures were utilized in more than half of the studies (*n* = 121, 54%) and mostly used to validate wearable data. Findings from these evaluations, such as those assessing correlation between data types, were highly variable. PROMs in these studies included measures of condition-specific health (e.g., Hip Disability and Osteoarthritis Outcome Score, HOOS) (*n* = 86, 71%), general health and quality of life (e.g., Patient Reported Outcome Measurement Information System (PROMIS)-Global, RAND 36-Item Short Form Health Survey) (*n* = 54, 45%), and psychosocial factors (e.g., PHQ-9) (*n* = 6, 5%). A single publication could utilize more than one PROM; thus, the values are higher than the total 121 of publications.

## 4. Discussion

Digital phenotyping and PGHD has been studied in a range of surgical contexts. Smartphones and wearable sensors have been used to capture an array of activity/mobility, biometric, and communication-related data. Studies have been conducted to establish the feasibility of these technologies to gather information from patients, while also assessing the potential for clinically meaningful functions, such as tracking and monitoring change in health status, decision support, and prediction of health outcomes. Our findings should be considered in light of some limitations.

### 4.1. Limitations

Firstly, scoping reviews encompassing broad concepts that generate large numbers of citations may be prone to human error where investigators inadvertently miss relevant articles. Further, where there are multiple investigators performing screening, there is a risk of alternative interpretations of abstracts. To mitigate this, we commenced screening following independent review and group discussion of a common set of articles, before proceeding with screening in batches and regular check-ins to query any concerns, share ideas, reach consensus, and resolve any disputes as necessary. Second, for speed, only two investigators were involved in developing the initial data charting system. A wider group discussion could have generated additional elements for consideration at the outset. Nevertheless, ample opportunities were built into our process for implementing ideas, new concepts, and iterating the data extraction chart. Third, given the heterogeneity of the articles and intention to encompass studies focused on technical feasibility as well as original research, it was challenging to identify a universal tool to appraise the quality and validity of the studies. Finally, while we aimed to comprehensively categorize the wide variety of devices among these studies, as none of the investigators were technologists, there may have been some degree of error in taxonomy and classification, especially among the commercial- and research-grade wearable/sensors. This may have been further complicated by the proprietary names for the devices which could have varied by geographical region or changed as technologies evolved.

Through the process of our full-text review, we identified three spheres of insights: clinical, technological/data, and interpersonal spheres, with future scopes of work required to realize the translation of personalized digital technologies from the research bench to surgical care [10].

### 4.2. Clinical Sphere

Authors have categorized surgical applications of wearable technologies into providing augmentative functions (the provision of information in real time for surgeons during clinical or surgical encounters, e.g., head-up displays on glasses), assistive functions (the use of wearables to replace physical tasks, e.g., gesture control of electronic systems while scrubbed for surgery), and assessment functions (i.e., objective measurement of clinical outcomes and disease severity, e.g., tracking mobility data and walking tolerance in degenerative musculoskeletal conditions) [24]. Wearable technologies can overlap to varying extents among these functions and be positioned at differing points along the continuum of surgical centeredness versus patient centeredness [24].

In this scoping review, beyond studies demonstrating technical feasibility alone, most studies involved the assessment function—commonly tracking and monitoring of activity and biometric data. Fewer studies involved prediction, risk profiling, surgical optimization, diagnostic processes, development of new interventions and care delivery models, shared decision making, decision support and targeted treatment selection, and recovery and rehabilitation support, e.g., gamification [25]. Personal digital technologies capturing PGHD were most commonly applied in the context of orthopaedics and neurosurgery. Applications mostly involved wearable motion sensors in populations with chronic musculoskeletal conditions, such as advanced osteoarthritis requiring total joint replacement [26,27,28,29]. In the context of musculoskeletal health in general, activity/mobility data (from accelerometers and GPS), communication data (text and telephone logs, screen time), and self-reported pain (phone-based visual analogue surveys) have been used to predict outcomes of care for spinal conditions [30]. Further, mobility metrics (gait speed using accelerometer and gyroscope sensors) from wearable sensors have been associated with health outcomes, including activities of daily life in older adults [31].

Beyond surgery, the concept of digital phenotyping has been extensively applied in the mental health arena for objective continuous generation of data points representing activities, cognitions, and behaviors (e.g., self-evaluated mood, daily steps, call durations, text frequency, psychosocial PROMs) in the management of a conditions including depression, anxiety, bipolar disease, schizophrenia and monitoring suicidal risk [32,33,34,35,36,37,38,39,40,41,42,43,44,45,46].

Digital phenotyping has also captured recovery metrics and physical activity in non-operative spinal care [18], augmented neurological care [47,48], signaled cardiovascular risk [49], characterized loneliness and social isolation [49], and been used to develop behavioral change interventions [50]. In relation to the point of application along the surgical pathway, personal digital devices have established baseline function [51,52,53], enabled advanced monitoring of biometric data during the perioperative phase/acute recovery phase [54], and tracked progress during postoperative recovery [25].

#### Future Scope

While there are a wide range of clinical applications, directions for further work and surgical use cases involving digital phenotyping can be summarized as (i) enhanced recovery monitoring, (ii) improving decision making, and (iii) surgical optimization (including optimization/prehabilitation). Further studies are also needed to understand the ability of PGHD to segment patient populations during the care cycle without stigmatizing the individual, define postoperative recovery trajectories, and assess the association of passive PGHD with PROMs.

As PGHD commonly involves activity-related metrics, there seems a natural opportunity to expand this form of measurement in orthopaedics to assess the association with PROMs capturing physical function, especially considering the direct impact of common conditions, such as osteoarthritis and fractures, and interventions, such as total joint replacement surgery and fracture fixation, on physical activities and mobility.

In relation to psychometric evaluation (i.e., assessment of validity, reliability, responsiveness, reproducibility, feasibility and user-friendliness), the same level of rigor applied to testing PROMs should be applied to passive PGHD. Full scale adoption of this technology across different surgical settings also requires forecasting of barriers and pitfalls related to surgical quality and safety, alongside the ethical, privacy, and legal considerations related to the use of this technology [18,55].

### 4.3. Technological/Data Sphere

Personal digital technologies such as smartphones—mobile devices used for core phone functions (voice calls, text messaging) and computing functions (wider software, internet, e.g., web browsing, mobile broadband, and multi-media functionality, e.g., gaming, music, video, cameras)—and wearable sensors (wearables)—small electronic devices embedded into items possessing computational ability that interface with the body—are now ubiquitous across the consumer market [3,24].

We categorized the technologies in this review into smartphone (e.g., Apple iPhone algorithm), consumer-grade wearables (e.g., Fitbit, Apple Watch, Garmin, Microsoft Band, Samsung Gear, Xiaomi MiBand, Huawei Band), and research-grade wearables and sensors (e.g., SenseWear Armband, ActivPal Monitors, Stepwatch Activity Monitor, DynaPort ADL monitor, ActiGraph GT3x Activity Monitor). There were no studies involving sensors embedded into other personal items, e.g., clothing, accessories such as contact lenses, in this review [3,24].

A rapidly evolving combination of sensors, displays, processors and storage memory, and interconnected software and computer algorithms are accelerating the collection, filtering, processing, interpretation, and visualization of an individual’s interactions with their environment from raw data [24]. In this review, we map an array of these generated data points and categorize them into an “Activity-Biometrics-Communication framework of digital biomarkers” from personal digital devices (Figure 6). The fast pace of this evolution is being fueled by developments in advanced technologies such as artificial intelligence and machine learning (especially around anomaly detection), increasing analytic capabilities alongside advances in collection and processing power. Increase in technical development has been matched by the explosion of scientific work in this field over the last twenty years. This growth may in part be due to the fast-paced release of wearable technologies in the health and fitness consumer market: FitBit releasing their first activity tracker and wearable technologies in 2014; Apple releasing the AppleWatch in 2015; and Garmin releasing the Forerunner 101 back in 2003.

Interestingly, the majority of studies in this review utilized research-grade technologies, despite most of the development, distribution, and sales occurring in the commercial sector. This raises questions around the availability, translation, and scalability of technologies developed and tested at the research bench, and whether such devices serve as appropriate benchmarks for testing commercial-grade devices. In contrast, the proprietary nature of the technology behind commercial-grade devices also warrants further discussion around standardization and scalability. How can health care professionals be sure that the summary statistics from different devices are measuring the same health domain? The heterogeneity throughout PGHD methodology provides a major challenge for scaling solutions.

#### Future Scope

Further work is needed around a data infrastructure and defining platforms necessary to support multiple forms of PGHD. The development of Fast Healthcare Interoperability Resources (FHIR), smart marker capabilities, and application programming interfaces (APIs) by the clinical informatics community should support the interoperability of personal digital technologies and applications in existing IT infrastructures as they become standardized within care pathways.

Infrastructure also depends on standardization of this technology, including the validation, testing, refinement, and standardizing the algorithms behind personal digital devices. The issue of standardization is particularly relevant when considering health domains derived from proprietary mathematical models, such as sleep quality. Greater clarity is needed around whether the raw data aligns with the processed data and whether these metrics are measuring what they claim to represent.

Validation of this technology should also include active PGHD, such as PROMs, and understanding the extent to which patient-reported outcomes can and/or should be used as comparators and benchmarks for passive PGHD. Extensive cycles of testing are needed to establish whether passive PGHD from personal digital devices can one day be used as standalone measures of health outcomes or be used side by side with active measurements.

Robust and transparent set of IT governance standards are required to optimize interoperability and reproducibility. In a broader sense, a strategic approach is needed to contend with the rate of technological advancement versus rate of adoption in existing health care settings. Further work should also evaluate the challenges around distribution, access to technology and costs.

### 4.4. Interpersonal Sphere

While digital phenotyping provides a rich source of PGHD to support the optimal delivery of surgical care, the nature of data captured using personal digital technologies (e.g., how many texts they wrote or how long they spent talking to friends and family; how long they took in moving from place to place); may further humanize the interaction between patients and clinicians [11,12,56]. This interaction contrasts with legacy electronic health record (EHR) systems and systems actively collecting PROMs (via in-person, digital and telephone assessment), that have led to patient and physician burden, burnout, inefficiencies, and distance between patients and providers.

A key aspect of humanizing the technology behind digital phenotyping requires an exploration of patient and professional perspectives around its acceptability, including sensitives and stigmas that may be associated with this form of data. Further, we need to understand how patients and professionals think and act in response to active and passive data capture, feedback, and visualization of metrics in relation to an individual’s condition. Interestingly, early work assessing personal digital data in the mental health arena has shown most patients (in outpatient settings) are happy to share social media and passive smartphone data [20,57]. In contrast, authors have also shown populations have been very apprehensive about actively reporting data summaries from their wearables with concerns around data privacy [18,58].

#### Future Scope

Further work is needed in surgical settings around the willingness of patients to have their personal passive data shared and utilized for their care [57]. Studies should also investigate the influence of this exposure on performance metrics and outcomes, as well as the way this data shapes the relationship with clinicians [15,59]. The aspect of data overload and data fatigue for clinicians should also be explored. When it comes to multi-disciplinary care and care spanning primary and secondary care, we should aim to define ownership and responsibility for the data.

The successful adoption of digital phenotyping requires an interdisciplinary approach involving co-collaboration and co-development of innovations between stakeholders in health care and digital health (patients and families, health care professionals, medical device industry, researchers, designers, technologists, bioengineers and scientists).

Future work should also explore considerations for integrating PGHD and digital phenotyping into existing patient pathways within and outside the walls of hospitals and clinics. The minimum level of technology required to integrate this technology should be defined alongside an understanding of the relative advantages of data generated for health systems at the clinical, institutional, network, and policy level.

## 5. Conclusions

Widescale adoption and use of smartphone and wearable technologies in the consumer and surgical health care sector has sparked opportunities to provide a digital phenotype for patients that aims to reflect their physical ability, cognition, social interaction and behavior in free-living settings. Active and passive data generated from sensors within these devices provide a nuanced view of patient outcomes for surgical conditions both alone and in combination with other data elements. While the ubiquity of such personal digital devices across society averts the need to introduce further technology, substantial further work is needed in relation to technological (data collection and analysis), clinical (standardized integration into workflows) and interpersonal (impact on patient–professional relationship) spheres of research and development. As technological, clinical, and interpersonal considerations unfold in this fast-moving space, more sophisticated ways of modelling themes, such as natural language processing of scientific and technical resources, can be used to better understand these elements [60,61]. Digital phenotyping offers an advanced understanding of human behavior and promises to drive objective, scalable, time sensitive, cost-effective, and reproducible digital outcome measurement for improving routine surgical care.

## Figures and Tables

**Table 1:** Summary of number of publications within country, surgical specialty, pathway phase, data type, and function categories.

	Category	Number of Publications
Country	USA	74
	UK	23
	Netherlands	21
	Australia	17
	Germany	14
	Switzerland	10
	Canada	7
	Spain	7
	Japan	6
	Italy	5
	South Korea	5
	Belgium	4
	Norway	4
	Sweden	4
	Brazil	3
	Denmark	3
	Taiwan	3
	China	2
	Greece	2
	Finland	1
	France	1
	Israel	1
	Portugal	1
	Romania	1
	Serbia	1
	South Africa	1
	Thailand	1
	Turkey	1
	Ukraine	1
Surgical Specialty	Bariatric	10
	Breast	1
	Cardiothoracic	15
	Colorectal	2
	General	13
	Neurosurgery	24
	Obstetrics/Gynecology	2
	Ophthalmology	3
	Oromaxillofacial	6
	Orthopaedics	129
	Surgical Oncology	11
	Transplant	7
	Urologic	1
	Vascular	4
Pathway Phase	Post	171
	Peri	4
	Pre, Post	36
	Pre, Peri, Post	2
	Peri, Post	1
	Pre	10
Data Type	Activity	122
	Biometrics	59
	Communication	0
	Activity, Biometrics	41
	Activity, Communication	2
Function	Feasibility	61
	Tracking or Monitoring	82
	Prediction	18
	Risk Profiling	8
	Optimization	18
	Feasibility, Tracking or Monitoring	10
	Feasibility, Prediction	1
	Feasibility, Risk Profiling, Prediction	1
	Feasibility, Tracking or Monitoring, Prediction	3
	Feasibility, Tracking or Monitoring, Risk Profiling	2
	Risk Profiling, Prediction	1
	Risk Profiling, Prediction, Optimization	1
	Tracking or Monitoring, Optimization	5
	Tracking or Monitoring, Prediction	6
	Tracking or Monitoring, Risk Profiling	5
	Tracking or Monitoring, Risk Profiling, Optimization	1
	Tracking or Monitoring, Risk Profiling, Prediction	1

## Appendix A

**Table A1:** PRISMA-Scoping Review (ScR) Checklist Preferred Reporting Items for Systematic reviews and Meta-Analyses extension for Scoping Reviews (PRISMA-ScR) Checklist.

SECTI.ON	ITEM	PRISMA-ScR CHECKLIST ITEM	REPORTED ON PAGE
**TITLE**			
Title	1	Identify the report as a scoping review.	1
**ABSTRACT**			
Structured summary	2	Provide a structured summary that includes (as applicable): background, objectives, eligibility criteria, sources of evidence, charting methods, results, and conclusions that relate to the review questions and objectives.	2
**INTRODUCTION**			
Rationale	3	Describe the rationale for the review in the context of what is already known. Explain why the review questions/objectives lend themselves to a scoping review approach.	3
Objectives	4	Provide an explicit statement of the questions and objectives being addressed with reference to their key elements (e.g., population or participants, concepts, and context) or other relevant key elements used to conceptualize the review questions and/or objectives.	3
**METHODS**			
Protocol and registration	5	Indicate whether a review protocol exists; state if and where it can be accessed (e.g., a Web address); and if available, provide registration information, including the registration number.	4
Eligibility criteria	6	Specify characteristics of the sources of evidence used as eligibility criteria (e.g., years considered, language, and publication status), and provide a rationale.	4
Information sources	7	Describe all information sources in the search (e.g., databases with dates of coverage and contact with authors to identify additional sources), as well as the date the most recent search was executed.	4
Search	8	Present the full electronic search strategy for at least one database, including any limits used, such that it could be repeated.	4; Appendix B
Selection of sources of evidence	9	State the process for selecting sources of evidence (i.e., screening and eligibility) included in the scoping review.	4
Data charting process	10	Describe the methods of charting data from the included sources of evidence (e.g., calibrated forms or forms that have been tested by the team before their use, and whether data charting was done independently or in duplicate) and any processes for obtaining and confirming data from investigators.	5
Data items	11	List and define all variables for which data were sought and any assumptions and simplifications made.	5
Critical appraisal of individual sources of evidence	12	If done, provide a rationale for conducting a critical appraisal of included sources of evidence; describe the methods used and how this information was used in any data synthesis (if appropriate).	n/a; Comment on page 5.
Synthesis of results	13	Describe the methods of handling and summarizing the data that were charted.	6
**RESULTS**			
Selection of sources of evidence	14	Give numbers of sources of evidence screened, assessed for eligibility, and included in the review, with reasons for exclusions at each stage, ideally using a flow diagram.	6
Characteristics of sources of evidence	15	For each source of evidence, present characteristics for which data were charted and provide the citations.	6
Critical appraisal within sources of evidence	16	If done, present data on critical appraisal of included sources of evidence (see item 12).	n/a
Results of individual sources of evidence	17	For each included source of evidence, present the relevant data that were charted that relate to the review questions and objectives.	6,7
Synthesis of results	18	Summarize and/or present the charting results as they relate to the review questions and objectives.	6,7
**DISCUSSION**			
Summary of evidence	19	Summarize the main results (including an overview of concepts, themes, and types of evidence available), link to the review questions and objectives, and consider the relevance to key groups.	7–11
Limitations	20	Discuss the limitations of the scoping review process.	7
Conclusions	21	Provide a general interpretation of the results with respect to the review questions and objectives, as well as potential implications and/or next steps.	11
FUNDING			
Funding	22	Describe sources of funding for the included sources of evidence, as well as sources of funding for the scoping review. Describe the role of the funders of the scoping review.	11

JBI = Joanna Briggs Institute; PRISMA-ScR = Preferred Reporting Items for Systematic reviews and Meta-Analyses extension for Scoping Reviews.

## Appendix B

Appendix B. Search strategy for PubMed involving terms related to “digital phenotyping and PGHD” (concept A), “outcome measurement” (concept B), and “surgical care” (concept C)

(“Digital phenotyp*”[Title/Abstract] OR “Wearable device*”[Title/Abstract] OR “Wearable motions sensor*”[Title/Abstract] OR “Wearable motion sensor*”[Title/Abstract] OR “Wearable motion sensing”[Title/Abstract] OR “Wearable sensor*”[Title/Abstract] OR “Wearable camera*”[Title/Abstract] OR “Wearable technolog*”[Title/Abstract] OR “Wearable electronic device*”[Title/Abstract] OR “Wearable activity monitor*”[Title/Abstract] OR “Wearable activity track*”[Title/Abstract] OR “Wearable activity device*”[Title/Abstract] OR “Wearablesensor technolog*”[Title/Abstract] OR “Wearable tracking system*”[Title/Abstract] OR Pedometer*[Title/Abstract] OR “Inertial motion sensor*” [Title/Abstract] OR “Sensor technolog*”[Title/Abstract] OR “Home sensing technolog*”[Title/Abstract] OR “Mobile health technolog*”[Title/Abstract] OR “Mobile technolog*”[Title/Abstract] OR “Activity monitor*”[Title/Abstract] OR Electrogoniomet*[Title/Abstract] OR “Strain gauges based sensor*”[Title/Abstract] OR “Textile piezoresistive sensor*”[Title/Abstract] OR “Wrist-worn activity monitor*”[Title/Abstract] OR “Activity-tracking wristband*”[Title/Abstract] OR “Activity tracker wristband*”[Title/Abstract] OR “Fitness tracker*”[Title/Abstract] OR “Fitness tracking”[Title/Abstract] OR “Commercial activity tracker*”[Title/Abstract] OR Fitbit*[Title/Abstract] OR “Apple watch*”[Title/Abstract] OR “Smart phone*”[Title/Abstract] OR “Smart device*”[Title/Abstract] OR Smartphone*[Title/Abstract] OR “Smartphone sensor*”[Title/Abstract] OR “Smartphone acceleromet*”[Title/Abstract]OR “Embedded sensor*”[Title/Abstract] OR “Personal digital device*”[Title/Abstract] OR “Digital device”[Title/Abstract] OR “Tracking device*”[Title/Abstract] OR “Human motion tracking system*”[Title/Abstract] OR “Wireless sensor*”[Title/Abstract] OR “Inertial sensor*”[Title/Abstract] OR “Inertial navigation system*”[Title/Abstract] OR Garmen[Title/Abstract] OR “GPS system*”[Title/Abstract] OR “Wearable Electronic Devices”[Mesh:NoExp] OR “Smartphone”[Mesh] OR “Fitness Trackers”[Mesh])

AND

(“Outcome Assessment, Health Care”[Mesh] OR “Patient Outcome Assessment”[Mesh] OR “Patient Reported Outcome Measures”[Mesh] OR “Patient Satisfaction”[Mesh] OR “Program Evaluation”[Mesh] OR “Process Assessment, Health Care”[Mesh] OR “Outcome and Process Assessment, Health Care”[Mesh] OR “Treatment Outcome”[Mesh] OR “Recovery of Function”[Mesh] OR “Patient Readmission”[Mesh] OR “Patient Discharge”[Mesh] OR “Self Care”[Mesh] OR “Patient Compliance”[Mesh] OR “Medication Adherence”[Mesh] OR “Quality of Life”[Mesh] OR “Fatal Outcome”[Mesh] OR “Activities of Daily Living”[Mesh] OR “Patient Acceptance of Health Care”[Mesh] OR “Treatment Adherence and Compliance”[Mesh] OR “Treatment Refusal”[Mesh] OR Outcome[Title/Abstract] OR outcomes[Title/Abstract] OR “Patient experience”[Title/Abstract] OR “Patient satisfaction”[Title/Abstract] OR “Patient expectation”[Title/Abstract] OR “Patient expectations”[Title/Abstract] OR “Process evaluation”[Title/Abstract] OR “Process assessment”[Title/Abstract] OR “Process measure”[Title/Abstract] OR “Process measurement”[Title/Abstract] OR “Program evaluation”[Title/Abstract] OR “Program assessment”[Title/Abstract] OR “Patient reported experience”[Title/Abstract] OR “Impact on patient”[Title/Abstract] OR “Surgical outcome”[Title/Abstract] OR “surgical outcomes”[Title/Abstract] OR “Financial cost”[Title/Abstract] OR “Economic impact”[Title/Abstract] OR “Economics”[Title/Abstract] OR “costs”[Title/Abstract] OR “Healthcare cost”[Title/Abstract] OR “Length of stay”[Title/Abstract] OR “Patient discharge”[Title/Abstract] OR “Complications”[Title/Abstract] OR “Readmission”[Title/Abstract] OR “readmissions”[Title/Abstract] OR “Emergency department visit”[Title/Abstract] OR “ED visit”[Title/Abstract] OR “Postoperative hospital visit”[Title/Abstract] OR “Postoperative care”[Title/Abstract] OR “Recovery”[Title/Abstract] OR “Self-management”[Title/Abstract] OR “Self-care”[Title/Abstract] OR “Self care”[Title/Abstract] OR “Treatment adherence”[Title/Abstract] OR “Medication adherence”[Title/Abstract] OR “Non-adherence”[Title/Abstract] OR “Follow-up visit”[Title/Abstract] OR “Post-surgical visit”[Title/Abstract] OR “Compliance”[Title/Abstract] OR “Non-compliance”[Title/Abstract] OR “Fear”[Title/Abstract] OR “Quality of life”[Title/Abstract] OR “Clinical effectiveness”[Title/Abstract] OR “Treatment effectiveness”[Title/Abstract] OR “Treatment efficacy”[Title/Abstract] OR “Clinical efficacy”[Title/Abstract] OR “Activities of Daily Living”[Title/Abstract])

AND

(Surger*[Title/Abstract] OR surgical[Title/Abstract] OR surgeon*[Title/Abstract] OR Operate[Title/Abstract] OR operative[Title/Abstract] OR operation[Title/Abstract] OR Gynecolog*[Title/Abstract] OR Neurosurg*[Title/Abstract] OR Obstetric*[Title/Abstract] OR Ophthalmolog*[Title/Abstract] OR Orthopedic*[Title/Abstract] OR orthopaedic*[Title/Abstract] OR Otolaryngolog*[Title/Abstract] OR Neurotolog*[Title/Abstract] OR Traumatolog*[Title/Abstract] OR Urolog*[Title/Abstract] OR Abdominoplasty[Title/Abstract] OR Ablation[Title/Abstract] OR Abortion[Title/Abstract] OR Acetabuloplasty[Title/Abstract] OR Acetabuloplasty[Title/Abstract] OR Adenoidectomy[Title/Abstract] OR Adrenalectomy[Title/Abstract] OR Amputation[Title/Abstract] OR Anastomosis[Title/Abstract] OR Apicoectomy[Title/Abstract] OR Appendectomy[Title/Abstract] OR Arthrodesis[Title/Abstract] OR Arthroplasty[Title/Abstract] OR Arthroscopy[Title/Abstract] OR “Biliopancreatic Diversion” [Title/Abstract] OR Biopsy[Title/Abstract] OR “Blalock-Taussig procedure”[Title/Abstract]OR Blepharoplasty[Title/Abstract] OR “Bone Lengthening” [Title/Abstract] OR Bypass[Title/Abstract] OR Castration[Title/Abstract] OR Cautery[Title/Abstract] OR Cementoplasty[Title/Abstract] OR “Cervical Cerclage”[Title/Abstract] OR “Cerebral Decortication”[Title/Abstract] OR “Cerebral Revascularization”[Title/Abstract] OR

Cervicoplasty[Title/Abstract] OR Cholecystostomy[Title/Abstract] OR Choledochostomy[Title/Abstract] OR Circumcision[Title/Abstract] OR Colectomy[Title/Abstract] OR Colposcopy[Title/Abstract] OR Colpotomy[Title/Abstract] OR Conization[Title/Abstract] OR Craniotomy[Title/Abstract] OR Cryosurgery[Title/Abstract] OR Culdoscopy[Title/Abstract] OR Curettage[Title/Abstract] OR Cystostomy[Title/Abstract] OR Dacryocystorhinostomy[Title/Abstract] OR Debridement[Title/Abstract] OR Decompression[Title/Abstract] OR “Obstetric delivery”[Title/Abstract] OR Denervation[Title/Abstract] OR Dilatation[Title/Abstract] OR Diskectomy[Title/Abstract] OR Dissection[Title/Abstract] OR Electrosurgery[Title/Abstract] OR Endoscopy[Title/Abstract] OR “Endovascular Procedures”[Title/Abstract] OR Enterostomy[Title/Abstract] OR Esophagectomy[Title/Abstract] OR Esophagoplasty[Title/Abstract] OR Esophagostomy[Title/Abstract] OR “Eye Enucleation”[Title/Abstract] OR “Eye visceration”[Title/Abstract] OR Fasciotomy[Title/Abstract] OR Fetoscopy[Title/Abstract] OR Foraminotomy[Title/Abstract] OR Fracture Fixation[Title/Abstract] OR Fundoplication[Title/Abstract] OR Gastrectomy [Title/Abstract] OR Gastroenterostomy[Title/Abstract] ORGastropexy[Title/Abstract] OR Gastroplasty[Title/Abstract] OR Gastrostomy[Title/Abstract] OR Gingivectomy[Title/Abstract] OR Gingivoplasty[Title/Abstract] OR Glossectomy[Title/Abstract] OR “Guided Tissue Regeneration”[Title/Abstract] OR “Heller Myotomy”[Title/Abstract] OR Hemofiltration[Title/Abstract] OR Hemoperfusion[Title/Abstract] OR Hemorrhoidectomy[Title/Abstract] OR Hepatectomy[Title/Abstract] OR Herniorrhaphy[Title/Abstract] OR Hypophysectomy[Title/Abstract] OR Hysteroscopy[Title/Abstract] OR Hysterotomy[Title/Abstract] OR Implantation[Title/Abstract] OR Iridectomy[Title/Abstract] OR “Jaw Fixation”[Title/Abstract] OR Keratectomy[Title/Abstract] OR Laminectomy[Title/Abstract] OR Laminoplasty[Title/Abstract] OR Laparotomy[Title/Abstract] OR Laryngectomy[Title/Abstract] OR Laryngoplasty[Title/Abstract] OR Laryngoscopy[Title/Abstract] OR “Laser Therapy”[Title/Abstract] OR Ligation[Title/Abstract] OR “Light Coagulation”[Title/Abstract] OR “Limb Salvage”[Title/Abstract] OR Lipectomy[Title/Abstract] OR Lithotripsy[Title/Abstract] OR Lobectomy[Title/Abstract] OR “Lymph Node Excision”[Title/Abstract] OR Mammaplasty[Title/Abstract] OR “Mandibular Advancement”[Title/Abstract] OR Mastectomy[Title/Abstract] OR “Maxillofacial Prosthesis Implantation”[Title/Abstract] OR Mediastinoscopy[Title/Abstract] OR Meniscectomy[Title/Abstract] OR Metastasectomy[Title/Abstract] OR Microsurgery[Title/Abstract] OR “Middle Ear Ventilation”[Title/Abstract] OR Mohs[Title/Abstract] OR Morcellation[Title/Abstract] OR Myotomy[Title/Abstract] OR “Percutaneous Nephrostomy”[Title/Abstract] OR Neuroendoscopy[Title/Abstract] OR “Ophthalmologic Orbit Evisceration”[Title/Abstract] OR “Orthopedic Procedures”[Title/Abstract] OR “Ossicular Replacement”[Title/Abstract] OR Osteotomy[Title/Abstract] OR Ostomy[Title/Abstract] OR Pallidotomy[Title/Abstract] OR Pancreaticoduodenectomy[Title/Abstract] OR Pancreaticojejunostomy[Title/Abstract] OR Paracentesis[Title/Abstract] OR Parathyroidectomy[Title/Abstract] OR “Pelvic Exenteration”[Title/Abstract] OR Phacoemulsification[Title/Abstract] OR Pharyngectomy[Title/Abstract] OR Pharyngostomy[Title/Abstract] OR Photopheresis[Title/Abstract] OR Piezosurgery[Title/Abstract] OR Pinealectomy[Title/Abstract] OR Pneumonectomy[Title/Abstract] OR Portoenterostomy[Title/Abstract] OR Proctectomy [Title/Abstract] OR Psychosurgery[Title/Abstract] OR Pyloromyotomy[Title/Abstract] OR Reconstruction[Title/Abstract] OR Reperfusion[Title/Abstract] OR Replantation[Title/Abstract] OR Resection[Title/Abstract] OR Rhinoplasty[Title/Abstract] OR Salpingostomy[Title/Abstract] OR “Scleral Buckling”[Title/Abstract] OR Scleroplasty[Title/Abstract] OR Sclerostomy[Title/Abstract] OR Shunt[Title/Abstract] OR “Sinus Floor Augmentation”[Title/Abstract] OR Sphincterotomy[Title/Abstract] OR “Spinal Puncture”[Title/Abstract] OR Splenectomy[Title/Abstract] OR “Split-Brain

Procedure”[Title/Abstract] OR “Stereotaxic Techniques”[Title/Abstract] OR “Reproductive Sterilization”[Title/Abstract] OR Sternotomy[Title/Abstract] OR Symphysiotomy[Title/Abstract] OR Synovectomy[Title/Abstract] OR Tendon Transfer[Title/Abstract] OR Tenodesis[Title/Abstract] OR Tenotomy[Title/Abstract] OR Thoracoplasty[Title/Abstract] OR Thoracoscopy [Title/Abstract] OR Thoracostomy[Title/Abstract] OR Thoracotomy[Title/Abstract] OR Thymectomy[Title/Abstract] OR Thyroidectomy[Title/Abstract] OR “Tissue Expansion”[Title/Abstract] OR Tonsillectomy[Title/Abstract] OR “Tooth Extraction”[Title/Abstract] OR “Tooth Replantation”[Title/Abstract] OR Tracheostomy[Title/Abstract] OR Tracheotomy[Title/Abstract] OR Tracheotomy[Title/Abstract] OR Traction[Title/Abstract] OR Transplant[Title/Abstract] OR Transplantation[Title/Abstract] OR “Ulnar Collateral Ligament Reconstruction”[Title/Abstract] OR Ultrafiltration[Title/Abstract] OR Ureterostomy[Title/Abstract] OR vasectomy[Title/Abstract] OR Vasovasostomy[Title/Abstract] OR Vitrectomy[Title/Abstract] OR “Surgical Procedures, Operative”[Mesh] OR “Specialties, Surgical”[Mesh] OR “Surgeons”[Mesh])

## Appendix C

**Table A2:** Final Study Set including study characteristics, clinical characteristics, technology/data characteristics and functional characteristics.

Title	Author	Year	Country	Study Design	Surgical Specialty	Pathway Phase	Technology Type	Data Type	Function
Physical activity monitors can be successfully implemented to assess Perioperative activity in urologic surgery	Agarwal, D. K., et al.	2018	USA	Feasibility/Validity	Urologic	Pre, Post	CGW	Activity	F
Reliability of Physical Activity Measures During Free-Living Activities in People After Total Knee Arthroplasty	Almeida, G. J., et al.	2016	USA	Feasibility/Validity	Orthopaedics	Post	RGW	Activity	TM, O
Responsiveness of Physical Activity Measures Following Exercise Programs after Total Knee Arthroplasty	Almeida, G. J., et al.	2017	USA	Feasibility/Validity	Orthopaedics	Post	RGW	Activity	O
Validity of physical activity measures in individuals after total knee arthroplasty	Almeida, G. J., et al.	2015	USA	Feasibility/Validity	Orthopaedics	Post	RGW	Activity	F
Kinematic and clinical evaluation of shoulder function after primary and revision reverse shoulder prostheses	Alta, T. D., et al.	2011	Netherlands	Original Prospective	Orthopaedics	Post	RGW	Biometrics	TM
The active and passive kinematic difference between primary reverse and total shoulder prostheses	Alta, T. D., et al.	2014	Netherlands	Original Prospective	Orthopaedics	Post	RGW	Biometrics	O
Long-term clinical evaluation of the automatic stance-phase lock-controlled prosthetic knee joint in young adults with unilateral above-knee Amputation	Andrysek, J., et al.	2017	Canada	Original Prospective	Orthopaedics	Post	CGW	Activity, Biometrics	TM
Mobile Phone-Connected Wearable Motion Sensors to Assess Postoperative Mobilization	Appelboom, G., et al.	2015	USA	Original Prospective	Neurosurgery	Post	CGW	Activity	F
Monitoring activity of hip injury patients (MoHIP): a sub-study of the World Hip Trauma Evaluation observational cohort study	Armitage, L. C., et al.	2020	UK	Original Prospective	Orthopaedics	Post	CGW	Activity	TM
High Plantar Force Loading After Achilles Tendon Rupture Repair with Early Functional Mobilization	Aufwerber, S., et al.	2019	Sweden	Original Prospective	Orthopaedics	Post	CGW	Activity	P
Psychological factors are associated with return to Pre-injury levels of sport and physical activity after ACL reconstruction	Baez, S. E., et al.	2020	USA	Original Prospective	Orthopaedics	Post	CGW	Activity	TM
Feasibility of low-cost accelerometers in measuring functional recovery after major oncologic surgery	Barkley, R., et al.	2019	USA	Feasibility/Validity	Surgical Oncology	Pre, Post	CGW	Activity	F
Assessment of a SP app (Capstesia) for measuring pulse Pressure variation: agreement between two methods: A Cross-sectional study	Barrachina, B., et al.	2017	Spain	Feasibility/Validity	General Surgery	Peri	SP	Biometrics	F
Physical Activity, Quality of Life and Body Image of Candidates to Bariatric Surgery	Barreto, B. L. M., et al.	2018	Brazil	Original Prospective	Bariatric	Post	CGW	Activity	O
Cementless THA for the treatment of osteonecrosis at 10-year follow-up: have we improved compared to cemented THA?	Bedard, N. A., et al.	2013	USA	Original Prospective	Orthopaedics	Post	RGW	Activity	TM
Functional outcome analysis of operatively treated malleolar fractures	Belcher, G. L., et al.	1997	USA	Original Prospective	Orthopaedics	Post	CGW	Activity	TM
Changes in prospectively collected longitudinal patient-generated health data are associated with short-term patient-reported outcomes after total joint arthroplasty: a pilot study	Bendich, I., et al.	2019	USA	Original Prospective	Orthopaedics	Post	CGW	Activity	RP
Activity levels and polyethylene wear of patients 10 years Post hip replacement	Bennett, D., et al.	2008	UK	Original Cross-sectional	Orthopaedics	Post	CGW	Activity	RP
Geriatric rehabilitation after hip fracture. Role of body-fixed sensor measurements of physical activity	Benzinger, P., et al.	2014	Germany	Original Cross-sectional	Orthopaedics	Post	CGW	Activity	F
Postoperative quality-of-life assessment in patients with spine metastases treated with long-segment pedicle-screw fixation	Bernard, F., et al.	2017	France	Original Retrospective	Neurosurgery	Post	CGW	Activity	TM
What are the functional outcomes of endoprosthestic reconstructions after tumor resection?	Bernthal, N. M., et al.	2015	USA	Original Prospective	Surgical Oncology	Post	RGW	Activity, Biometrics	P
Pervasive wearable device for free tissue transfer monitoring based on advanced data analysis: clinical study report	Berthelot, M., et al.	2019	UK	Original Prospective	Breast	Peri	RGW	Biometrics	F
Machine Learning Algorithms Can Use Wearable Sensor Data to Accurately Predict Six-Week Patient-Reported Outcome Scores Following Joint Replacement in a Prospective Trial	Bini, S. A., et al.	2019	USA	Original Prospective	Orthopaedics	Post	CGW	Activity, Biometrics	P
Monitoring of Postoperative Bone Healing Using Smart Trauma-Fixation Device with Integrated Self-Powered Piezo-Floating-Gate Sensors	Borchani, W., et al.	2015	USA	Feasibility/Validity	Orthopaedics	Post	RGW	Biometrics	F
Cross-sectional assessment of daily physical activity in chronic obstructive Pulmonary disease lung transplant patients	Bossenbroek, L., et al.	2009	Netherlands	Original Cross-sectional	Transplant	Post	CGW	Activity, Biometrics	TM
Changes in physical activity and health-related quality of life during the first year after total knee arthroplasty	Brandes, M., et al.	2011	Germany	Original Prospective	Orthopaedics	Pre, Post	RGW	Activity, Biometrics	TM
Quantity versus quality of gait and quality of life in patients with osteoarthritis	Brandes, M., et al.	2008	Germany	Original Prospective	Orthopaedics	Pre, Post	RGW	Activity, Biometrics	F
Impact of a tailored activity counselling intervention during inpatient rehabilitation after knee and hip arthroplasty—an explorative RCT	Brandes, M., et al.	2018	Germany	Original Prospective	Orthopaedics	Post	RGW	Activity	O
Reliability of wireless monitoring using a wearable patch sensor in high-risk surgical patients at a step-down unit in the Netherlands: a clinical validation study	Breteler, M. J.M. M., et al.	2018	Netherlands	Original Prospective	General Surgery	Post	RGW	Activity, Biometrics	F
Are current wireless monitoring systems capable of detecting adverse events in high-risk surgical patients? A descriptive study	Breteler, M. J. M., et al.	2020	Netherlands	Original Cross-sectional	General Surgery	Post	RGW	Biometrics	F
Vital Signs Monitoring with Wearable Sensors in High-risk Surgical Patients: A Clinical Validation Study	Breteler, M. J. M., et al.	2020	Netherlands	Original Cross-sectional	General Surgery	Post	RGW	Biometrics	TM
Novel positioning sensor with real-time feedback for improved Postoperative positioning: pilot study in control subjects	Brodie, F. L., et al.	2017	USA	Original Prospective	Ophthalmology	Peri	CGW	Biometrics	F
Validity and reliability of measurements obtained with an “activity monitor” in people with and without a transtibial Amputation	Bussmann, H. B., et al.	1998	Netherlands	Feasibility/Validity	Orthopaedics	Post	RGW	Activity, Biometrics	F
Validity of the prosthetic activity monitor to assess the duration and spatio-temporal characteristics of prosthetic walking	Bussmann, J. B., et al.	2004	Netherlands	Feasibility/Validity	Orthopaedics	Post	RGW	Biometrics	F
Ambulatory accelerometry to quantify motor behaviour in patients after failed back surgery: a validation study	Bussmann, J. B., et al.	1998	Netherlands	Feasibility/Validity	Neurosurgery	Post	RGW	Activity, Biometrics	F
Inertial Sensor-Based Gait and Attractor Analysis as Clinical Measurement Tool: Functionality and Sensitivity in Healthy Subjects and Patients with Symptomatic Lumbar Spinal Stenosis	Byrnes, S. K., et al.	2018	Switzerland	Feasibility/Validity	Neurosurgery	Post	RGW	Biometrics	O
Cardiac Surgery Rehabilitation System (CSRS) for a Personalized Support to Patients	Caggianese, G., et al.	2017	Italy	Original Prospective	Cardiothoracic	Post	CGW	Activity	TM
Clinical evaluation of a mobile sensor-based gait analysis method for outcome measurement after knee arthroplasty	Calliess, T., et al.	2014	Germany	Feasibility/Validity	Orthopaedics	Pre, Post	RGW	Activity, Biometrics	F
Higher pyruvate levels after Achilles tendon rupture surgery could be used as a prognostic biomarker of an improved patient outcome	Capone, G., et al.	2020	Sweden	Original Prospective	Orthopaedics	Post	CGW	Activity	P
Wearable Technology-A Pilot Study to Define “Normal” Postoperative Recovery Trajectories	Carmichael, H., et al.	2019	USA	Original Prospective	General Surgery	Pre, Post	CGW	Activity	TM
Patterns of physical activity and sedentary behavior after Bariatric: an observational study	Chapman, N., et al.	2014	Australia	Original Prospective	Bariatric	Post	RGW	Activity, Biometrics	O
Data Collection and Analysis Using Wearable Sensors for Monitoring Knee Range of Motion after Total Knee Arthroplasty	Chiang, C. Y., et al.	2017	Taiwan	Original Prospective	Orthopaedics	Post	RGW	Activity	F
Feasibility and Preliminary Outcomes of a Physical Therapist-Administered Physical Activity Intervention After Total Knee Replacement	Christiansen, M. B., et al.	2019	USA	Feasibility/Validity	Orthopaedics	Post	CGW	Activity	F
An Assessment of Physical Activity Data Collected via a Smartphone App and a Smart Band in Breast Cancer Survivors: Observational Study	Chung, I. Y., et al.	2019	South korea	Original Prospective	Surgical Oncology	Post	SP, CGW	Activity, Biometrics	F
Inertial sensor-based measures of gait symmetry and repeatability in people with unilateral lower limb Amputation	Clemens, S., et al.	2020	USA	Original Prospective	Orthopaedics	Post	RGW	Activity	TM
Use of a wrist-mounted device for continuous outpatient physiologic monitoring after transsphenoidal surgery: a pilot study	Cole, T. S., et al.	2019	USA	Original Prospective	Oromaxillofacial	Post	CGW	Activity, Biometrics	F
Understanding the Capacity for Exercise in Post-Bariatric Patients	Coleman, K. J., et al.	2017	USA	Original Prospective	Bariatric	Post	CGW	Activity	F, TM
A multicomponent intervention to decrease sedentary time during hospitalization: a quasi-exPerimental pilot study	Conijn, D., et al.	2020	Netherlands	Original Prospective	Vascular, Transplantation	Post	CGW	Activity	F
Digital Phenotyping in Patients with Spine Disease: A Novel Approach to Quantifying Mobility and Quality of Life	Cote, D. J., et al.	2019	USA	Original Prospective	Neurosurgery	Post	SP	Activity, Communication	TM
Late effects of a brief psychological intervention in patients with intermittent claudication in a randomized clinical trial	Cunningham, M. A., et al.	2013	Australia	Original Prospective	Vascular	Post	Unknown	Activity	P
Daily Physical Activity in Total Hip Arthroplasty Patients Undergoing Different Surgical Approaches A Cohort Study	Engdal, M., et al.	2017	Norway	Original Prospective	Orthopaedics	Post	RGW	Activity, Biometrics	TM
Validation of the Fitbit Flex in an Acute Post-Cardiac Surgery Patient Population	Daligadu, J., et al.	2018	Canada	Feasibility/Validity	Cardiothoracic	Post	CGW	Activity	F
Association of Wearable Activity Monitors with Assessment of Daily Ambulation and Length of Stay Among Patients Undergoing Major Surgery	Daskivich, T. J., et al.	2019	USA	Original Prospective	Cardiothoracic, General Surgery, Bariatric	Post	CGW	Activity	F
Are patients with knee osteoarthritis and patients with knee joint replacement as physically active as healthy persons?	Daugaard, R., et al.	2018	Denmark	Original Prospective	Orthopaedics	Post	CGW	Activity	TM
Physical Activity Levels During Acute Inpatient Admission After Hip Fracture are Very Low	Davenport, S. J., et al.	2014	Australia	Original Cross-sectional	Orthopaedics	Pre, Post	RGW	Activity, Biometrics	TM
Feasibility of real-time location systems in monitoring recovery after major abdominal surgery	Dorrell, R. D., et al.	2017	USA	Original Prospective	General Surgery	Post	RGW	Activity	TM
Continuous Versus Intermittent Vital Signs Monitoring Using a Wearable, Wireless Patch in Patients Admitted to Surgical Wards: Pilot Cluster Randomized Controlled Trial	Downey, C., et al.	2018	UK	Feasibility/Validity	General Surgery	Post	RGW	Biometrics	F
Distribution of arm velocity and frequency of arm usage during daily activity: objective outcome evaluation after shoulder surgery	Duc, C., et al.	2013	Switzerland	Feasibility/Validity	Orthopaedics	Post	RGW	Biometrics	TM
Objective evaluation of cervical spine mobility after surgery during free-living activity	Duc, C., et al.	2013	Belgium	Feasibility/Validity	Neurosurgery	Post	RGW	Biometrics	TM
Ambulation monitoring of transtibial Amputation subjects with patient activity monitor versus pedometer	Dudek, N. L., et al.	2008	Canada	Feasibility/Validity	Orthopaedics	Post	CGW	Activity	F
Evaluating patients’ walking capacity during hospitalization for lung cancer resection	Esteban, P. A., et al.	2017	Spain	Original Cross-sectional	Cardiothoracic	Post	CGW	Activity	TM
Activity and socket wear in the Charnley low-friction arthroplasty	Feller, J. A., et al.	1994	Australia	Original Retrospective	Orthopaedics	Post	Unknown	Activity	TM
Physical activity monitoring: a responsive and meaningful patient-centered outcome for surgery, chemotherapy, or radiotherapy?	Ferriolli, E., et al.	2012	UK	Original Cross-sectional	General Surgery	Post	RGW	Activity	F
A feasibility study of an unsupervised, Pre-operative exercise program for adults with lung cancer	Finley, D. J., et al.	2020	USA	Feasibility/Validity	Cardiothoracic	Pre	CGW	Activity, Biometrics	F
Differences in Preferred walking speeds in a gait laboratory compared with the real world after total hip replacement	Foucher, K. C., et al.	2010	USA	Original Prospective	Orthopaedics	Post	RGW	Activity	TM
Pilot study of methods to document quantity and variation of independent patient exercise and activity after total knee arthroplasty	Franklin, P. D., et al.	2006	USA	Feasibility/Validity	Orthopaedics	Post	RGW	Activity	F, TM
Improvements in Objectively Measured Activity Behaviors Do Not Correlate with Improvements in Patient-Reported Outcome Measures Following Total Knee Arthroplasty	Frimpong, E., et al.	2020	South Africa	Original Prospective	Orthopaedics	Pre, Post	RGW	Activity	P
Prospective study of physical activity and quality of life in Japanese women undergoing total hip arthroplasty	Fujita, K., et al.	2013	Japan	Original Cross-sectional	Orthopaedics	Pre, Post	RGW	Activity	TM
Effects of cycle ergometer use in early mobilization following cardiac surgery: a randomized controlled trial	Gama Lordello, G. G., et al.	2020	Brazil	Original Prospective	Cardiothoracic	Post	CGW	Activity	P
Enhancing patient mobility following cesarean-delivery—the efficacy of an improved Postpartum protocol assessed with pedometers	Ganer Herman, H., et al.	2020	Israel	Original Prospective	Obstetrics/Gynecology	Post	CGW	Activity	P
Assessment and Post-Intervention recovery following surgery for Lumbar Disc Herniation based on objective gait metrics from wearable devices using the Gait Posture index: GPi™	Ghent, F., et al.	2020	Australia	Feasibility/Validity	Neurosurgery	Pre, Post	CGW	Activity, Biometrics	TM
Physical activity patterns of patients immediately after lumbar surgery	Gilmore, S. J., et al.	2019	Australia	Original Prospective	Neurosurgery	Post	RGW	Activity	P
Assessing the utility of an IoS application in the Perioperative care of spine surgery patients: the NeuroPath Pilot study	Glauser, G., et al.	2019	USA	Feasibility/Validity	Neurosurgery	Pre, Post	SP	Activity, Communication	F
A Step in the Right Direction: Body Location Determines Activity Tracking Device Accuracy in Total Knee and Hip Arthroplasty Patients	Goel, R., et al.	2020	USA	Original Prospective	Orthopaedics	Post	CGW	Biometrics	TM
Comparative study of the activity of total hip arthroplasty patients and normal subjects	Goldsmith, A. A., et al.	2001	UK	Original Prospective	Orthopaedics	Post	CGW	Activity	TM
CAPACITY: A physical activity self-management program for patients undergoing surgery for lung cancer, a phase I feasibility study	Granger, C. L., et al.	2018	Australia	Original Prospective	Cardiothoracic	Pre, Post	CGW	Activity	F
Accelerometery as a measure of modifiable physical activity in high-risk elderly Preoperative patients: a prospective observational pilot study	Grimes, L., et al.	2019	UK	Original Prospective	General Surgery	Pre	CGW	Activity	F, TM
Does the Femoral Head Size in Hip Arthroplasty Influence Lower Body Movements during Squats, Gait and Stair Walking? A Clinical Pilot Study Based on Wearable Motion Sensors	Grip, H., et al.	2019	Sweden	Feasibility/Validity	Orthopaedics	Post	RGW	Activity, Biometrics	F
Assessment of objective ambulation in lower extremity sarcoma patients with a continuous activity monitor: rationale and validation	Gundle, K. R., et al.	2014	USA	Feasibility/Validity	Surgical Oncology	Post	RGW	Activity	F
Remote Gait Analysis Using Wearable Sensors Detects Asymmetric Gait Patterns in Patients Recovering from ACL Reconstruction	Gurchiek, R. D., et al.	2019	USA	Original Cross-sectional	Orthopaedics	Post	RGW	Biometrics	F
Open-Source Remote Gait Analysis: A Post-Surgery Patient Monitoring Application	Gurchiek, R. D., et al.	2019	USA	Feasibility/Validity	Orthopaedics	Post	SP, RGW	Activity, Biometrics	F
Physical performance and self-report outcomes associated with use of passive, adaptive, and active prosthetic knees in persons with unilateral, transfemoral Amputation: Randomized crossover trial	Hafner, B. J. and R. L. Askew	2015	USA	Original Prospective	Orthopaedics	Post	RGW	Activity	TM
Using MEMS-based inertial sensor with ankle foot orthosis for telerehabilitation and its clinical evaluation in brain injuries and total knee replacement patients	Han, S. L., et al.	2016	Taiwan	Feasibility/Validity	Orthopaedics	Post	RGW	Activity	F
Do activity levels increase after total hip and knee arthroplasty?	Harding, P., et al.	2014	Australia	Original Prospective	Orthopaedics	Pre, Post	RGW	Activity	TM
Knee arthroplasty: a cross-sectional study assessing energy expenditure and activity	Hayes, D. A., et al.	2011	Australia	Original Cross-sectional	Orthopaedics	Pre, Post	RGW	Activity, Biometrics	P
Wearable Technology in the Perioperative Period: Predicting Risk of Postoperative Complications in Patients Undergoing Elective Colorectal	Hedrick, T. L., et al.	2020	USA	Original Prospective	Colorectal	Pre, Post	CGW	Activity	RP
Detecting Postural transitions: a robust wavelet-based approach	Hemmati, S. and E. Wade	2016	USA	Original Prospective	Orthopaedics	Post	RGW	Activity	F
Low validity of the Sensewear Pro3 activity monitor compared to indirect calorimetry during simulated free living in patients with osteoarthritis of the hip	Hermann, A., et al. (2014).	2014	Denmark	Original Cross-sectional	Orthopaedics	Pre, Post	RGW	Biometrics	F
Clinical outcome and physical activity measured with StepWatch 3 (TM) Activity Monitor after minimally invasive total hip arthroplasty	Holl, S., et al.	2018	Germany	Original Prospective	Orthopaedics	Pre, Post	RGW	Activity	TM
Interaction between physical activity and continuous-flow left ventricular assist device function in outpatients	Hu, S.X., et al.	2013	Australia	Original Prospective	Cardiothoracic	Post	RGW	Activity, Biometrics	TM, P
2009 Marshall Urist Young Investigator Award: how often do patients with high-flex total knee arthroplasty use high flexion?	Huddleston, J. I., et al.	2009	USA	Original Cross-sectional	Orthopaedics	Post	RGW	Activity	TM, P
Tri-axial accelerometer analysis techniques for evaluating functional use of the extremities	Hurd, W.J., et al.	2013	USA	Original Prospective	Orthopaedics	Pre	RGW	Activity	F
Patient-Reported and Objectively Measured Function Before and After Reverse Shoulder Arthroplasty	Hurd, W.J., et al.	2018	USA	Original Prospective	Orthopaedics	Post	RGW	Activity	F, TM
A Smart Assistance Solution for Remotely Monitoring the Orthopaedic Rehabilitation Process Using Wearable Technology: re.flex System	Ianculescu, M., et al.	2019	Romania	Feasibility/Validity	Orthopaedics	Post	CGW	Activity	F
Physical activity patterns and function 3 months after arthroscopic partial meniscectomy	Ilich, S.S., et al.	2012	Australia	Original Prospective	Neurosurgery	Post	RGW	Activity	TM
Objective evaluation of Postoperative changes in real-life activity levels in the Postoperative course of lumbar spinal surgery using wearable trackers	Inoue, M., et al.	2020	Japan	Original Prospective	Neurosurgery	Post	RGW	Activity	TM
HipGuard: A Wearable Measurement System for Patients Recovering from a Hip Operation	Iso-Ketola, P., et al.	2008	Finland	Feasibility/Validity	Orthopaedics	Post	RGW	Biometrics	F
Upright Time and Sit-To-Stand Transition Progression After Total Hip Arthroplasty: An Inhospital Longitudinal Study	Jeldi, A. J., et al.	2016	UK	Original Prospective	Orthopaedics	Post	RGW	Biometrics	TM
Metal ion concentrations after metal-on-metal hip arthroplasty are not correlated with habitual physical activity levels	Jelsma, J., et al.	2019	Netherlands	Original Prospective	Orthopaedics	Post	RGW	Activity	P
Association of Daily Step Count with the Prolonged Air Leak in Thoracic Surgery Patients	Kavurmaci, Ö., et al.	2020	Turkey	Original Cross-sectional	Cardiothoracic	Post	Unknown	Activity	P
The Usefulness of a Wearable Device in Daily Physical Activity Monitoring for the Hospitalized Patients Undergoing Lumbar Surgery	Kim, D. H., et al.	2019	South Korea	Original Prospective	Neurosurgery	Post	CGW	Activity, Biometrics	TM, P
Associations between physical activity and mental health among Bariatric surgical candidates	King, W. C., et al.	2013	USA	Original Cross-sectional	Bariatric	Pre	RGW	Activity	TM, RP, O
Seasonal Variation in Physical Activity among Preoperative Patients with Lung Cancer Determined Using a Wearable Device	Kong, S., et al.	2020	South Korea	Original Cross-sectional	Cardiothoracic	Pre	CGW	Activity	TM
Gamified 3D Orthopaedic Rehabilitation using Low Cost and Portable Inertial Sensors	Kontadakis, G., et al.	2017	Greece	Feasibility/Validity	Orthopaedics	Post	RGW	Activity	F
Relationship Between Physical Activity and Clinical Outcomes After ACL Reconstruction	Kuenze, C., et al.	2019	USA	Original Cross-sectional	Orthopaedics	Post	RGW	Activity	F, TM
Gait Pattern Recognition Using a Smartwatch Assisting Postoperative Physiotherapy	Kyritsis, A. I., et al.	2019	Switzerland	Original Prospective	Orthopaedics	Post	CGW	Biometrics	F
Gait Recognition with Smart Devices Assisting Postoperative Rehabilitation in a Clinical Setting	Kyritsis, A. I., et al.	2018	Switzerland	Feasibility/Validity	Orthopaedics	Post	CGW	Biometrics	F
Recovery of mobility after knee arthroplasty—Expected rates and influencing factors	Lamb, S. E. and H. Frost	2003	UK	Original Prospective	Orthopaedics	Post	RGW	Biometrics	TM
Physical activity is unrelated to cognitive performance in Pre-Bariatric patients	Langenberg, S., et al.	2015	Germany	Original Prospective	Bariatric	Pre	RGW	Activity, Biometrics	RP, P, O
Physical activity in daily life 1 year after lung transplantation	Langer, D., et al.	2009	Belgium	Original Prospective	Transplant	Post	RGW	Activity, Biometrics	TM
Predicting physical activity recovery after hip and knee arthroplasty? A longitudinal cohort study	Lebleu, J., et al.	2019	Belgium	Original Cross-sectional	Orthopaedics	Post	CGW	Activity	TM, P
iHandU: Towards the Validation of a Wrist Rigidity Estimation for Intraoperative DBS Electrode Position Optimization	Lopes, E. M., et al.	2019	Portugal	Feasibility/Validity	Neurosurgery	Peri	RGW	Biometrics	F
Adherence to a pedometer-based physical activity intervention following kidney transplant and impact on metabolic parameters	Lorenz, E. C., et al.	2015	USA	Original Prospective	Transplant	Post	CGW	Activity	F, TM, P
Financial Incentives and Health Coaching to Improve Physical Activity Following Total Knee Replacement: A Randomized Controlled Trial	Losina, E., et al.	2018	USA	Original Prospective	Orthopaedics	Post	CGW	Activity	F, TM, P
Fitbit step counts during inpatient recovery from cancer surgery as a Predictor of readmission	Low, C. A., et al.	2017	USA	Original Prospective	Surgical Oncology	Post	CGW	Activity	TM, RP
Is Activity Tracker-Measured Ambulation an Accurate and Reliable Determinant of Postoperative Quality of Recovery? A Prospective Cohort Validation Study	Massouh, F., et al.	2019	Canada	Original Prospective	Obstetrics/Gynecology	Post	CGW	Activity	F
Relationship between body mass index and activity in hip or knee arthroplasty patients	McClung, C. D., et al.	2000	USA	Original Cross-sectional	Orthopaedics	Post	Unknown	Activity	TM, RP
Patient-Generated Actigraphy Data as a Novel Outcomes Instrument in Carpal Tunnel Syndrome	McMahon, H. A., et al.	2020	USA	Original Prospective	Orthopaedics	Post	RGW	Activity, Biometrics	F
Use of the pedometer in the evaluation of the effects of rehabilitation treatment on deambulatory autonomy in patients with lower limb arthroplasty during hospital rehabilitation: long-term Postoperative outcomes	Melchiorri, G., et al.	2020	Italy	Original Cross-sectional	Orthopaedics	Post	CGW	Activity	F, TM
Physical Function and Pre-Amputation Characteristics Explain Daily Step Count after Dysvascular Amputation	Miller, M. J., et al.	2019	USA	Original Cross-sectional	Vascular	Post	RGW	Activity	F, TM, RP
Evaluation of respiratory status and mandibular movement after total temporomandibular joint replacement in patients with rheumatoid arthritis	Mishima, K., et al.	2003	Japan	Original Prospective	Oromaxillofacial	Pre, Post	RGW	Biometrics	F, TM
Real-Time Monitoring of Bone Fracture Recovery by Using Aware, Sensing, Smart, and Active Orthopedic Devices	Mišić, D., et al.	2018	Serbia	Feasibility/Validity	Orthopaedics	Post	RGW	Biometrics	F
Proposed objective scoring algorithm for assessment and intervention recovery following surgery for lumbar spinal stenosis based on relevant gait metrics from wearable devices: the Gait Posture index (GPi)	Mobbs, R. J., et al.	2019	Australia	Feasibility/Validity	Neurosurgery	Post	CGW	Activity, Biometrics	F, RP, P
Physical Activity Measured with Accelerometer and Self-Rated Disability in Lumbar Spine Surgery: A Prospective Study	Mobbs, R. J., et al.	2016	Australia	Original Prospective	Neurosurgery	Pre, Post	CGW	Activity, Biometrics	F, TM, RP
Outcome of the modified Lapidus procedure for hallux valgus deformity during the first year following surgery: A prospective clinical and gait analysis study	Moerenhout, K., et al.	2019	Switzerland	Original Prospective	Orthopaedics	Post	RGW	Biometrics	F, TM
Physical Function, Quality of Life, and Energy Expenditure During Activities of Daily Living in Obese, Post-Bariatric, and Healthy Subjects	Monteiro, F., et al.	2017	Brazil	Original Prospective	Bariatric	Post	RGW	Activity	F, TM, P
Towards a new Concept to the Neurological Recovery for Knee Stabilization after Anterior Cruciate Ligament Reconstruction Based on Surface Electrical Stimulation	Moreno, J. C., et al.	2008	Spain	Feasibility/Validity	Orthopaedics	Post	RGW	Biometrics	F
Duration and frequency of every day activities in total hip patients	Morlock, M., et al.	2001	Germany	Original Prospective	Orthopaedics	Post	RGW	Activity	F, TM
Physical performance in kidney transplanted patients: a study on desert trekking	Mosconi, G., et al.	2011	Italy	Original Prospective	Transplant	Post	RGW	Activity, Biometrics	TM
Identifying subgroups of community-dwelling older adults and their prospective associations with long-term knee osteoarthritis outcomes	Munugoda, I. P., et al.	2020	Australia	Original Prospective	Orthopaedics	Pre	CGW	Activity	TM, RP, P
High-grade rotatory knee laxity may be Predictable in ACL injuries	Musahl, V., et al.	2018	USA	Original Prospective	Orthopaedics	Pre	RGW	Biometrics	RP, P
The effect of patella resurfacing in total knee arthroplasty on functional range of movement measured by flexible electrogoniometry	Myles, C. M., et al.	2006	UK	Original Prospective	Orthopaedics	Post	RGW	Biometrics	TM, P
Knee joint functional range of movement prior to and following total knee arthroplasty measured using flexible electrogoniometry	Myles, C. M., et al.	2002	UK	Original Prospective	Orthopaedics	Pre, Post	RGW	Biometrics	TM
How Many Steps Per Day are Necessary to Prevent Postoperative Complications Following Hepato-Pancreato-Biliary Surgeries for Malignancy?	Nakajima, H., et al.	2020	Japan	Original Prospective	Surgical Oncology, General Surgery	Pre	RGW	Activity	RP
Assessment of Early Gait Recovery After Anterior Approach Compared to Posterior Approach Total Hip Arthroplasty: A Smartphone Accelerometer-Based Study	Nelms, N. J., et al.	2019	USA	Original Prospective	Orthopaedics	Pre, Post	SP	Activity, Biometrics	RP
Value of the average basal daily walked distance measured using a pedometer to Predict maximum oxygen consumption per minute in patients undergoing lung resection	Novoa, N. M., et al.	2011	Spain	Original Prospective	Cardiothoracic	Pre, Post	CGW	Activity	F, P
Influence of major Pulmonary resection on Postoperative daily ambulatory activity of the patients	Novoa, N., et al.	2009	Spain	Original Prospective	Cardiothoracic	Pre, Post	CGW	Activity, Biometrics	P
A prospective randomised double-blind study of functional outcome and range of flexion following total knee replacement with the NexGen standard and high flexion components	Nutton, R. W., et al.	2008	UK	Original Prospective	Orthopaedics	Post	RGW	Activity, Biometrics	TM
Does a mobile-bearing, high-flexion design increase knee flexion after total knee replacement?	Nutton, R. W., et al.	2012	UK	Original Prospective	Orthopaedics	Post	RGW	Biometrics	TM
Preoperative home-based physical therapy versus usual care to improve functional health of frail older adults scheduled for elective total hip arthroplasty: a pilot randomized controlled trial	Oosting, E., et al.	2012	Netherlands	Feasibility/Validity	Orthopaedics	Pre, Post	CGW	Activity	F
User Friendliness of a Wearable Visual Behavior Monitor for Cataract and Refractive Surgery	Pajic, B., et al.	2020	Switzerland	Original Prospective	Ophthalmology	Pre	CGW	Biometrics	F
Mandibular motion after closed and open treatment of unilateral mandibular condylar process fractures	Palmieri, C., et al.	1999	USA	Original Prospective	Oromaxillofacial	Post	RGW	Biometrics	TM
Using Smartphones to Capture Novel Recovery Metrics After Cancer Surgery	Panda, N., et al.	2020	USA	Original Prospective	Surgical Oncology	Post	SP	Activity	F
Wearable activity sensors and early pain after total joint arthroplasty	Patterson, J. T., et al.	2020	USA	Original Prospective	Orthopaedics	Post	CGW	Activity, Biometrics	TM, RP
Armband activity monitor data do not correlate with reported pain scores in patients receiving vertebroplasty	Peacock, J. G., et al.	2016	USA	Original Prospective	Neurosurgery	Post	RGW	Activity, Biometrics	TM, RP
Alteration and recovery of arm usage in daily activities after rotator cuff surgery	Pichonnaz, C., et al.	2015	Switzerland	Original Prospective	Orthopaedics	Post	RGW	Activity	TM
Objectively measured mobilisation is enhanced by a new behaviour support tool in patients undergoing abdominal cancer surgery	Porserud, A., et al.	2019	Sweden	Original Prospective	Surgical Oncology	Pre, Post	RGW	Activity	TM
Activity and affect: repeated within-participant assessment in people after joint replacement surgery	Powell, R., et al.	2009	UK	Original Prospective	Orthopaedics	Post	RGW	Activity	P
Continuous Digital Assessment for Weight Loss Surgery Patients	Ramirez, E., et al.	2020	USA	Original Prospective	Bariatric	Post	CGW	Biometrics	TM
Remote Patient Monitoring Using Mobile Health for Total Knee Arthroplasty: Validation of a Wearable and Machine Learning-Based Surveillance Platform	Ramkumar, P. N., et al.	2019	USA	Feasibility/Validity	Orthopaedics	Post	SP, RGW	Activity, Biometrics	TM
Walking, Sedentary Time and Health-Related Quality Life Among Kidney Transplant Recipients: An Exploratory Study	Raymond, J., et al.	2015	Canada	Original Cross-sectional	Transplant	Post	RGW	Activity, Biometrics	O
Dual Mode Gait Sonification for Rehabilitation After Unilateral Hip Arthroplasty	Reh, J., et al.	2019	Germany	Original Prospective	Orthopaedics	Post	RGW	Activity, Biometrics	TM
A prospective randomized comparison of the minimally invasive direct anterior and the transgluteal approach for primary total hip arthroplasty	Reichert, J. C., et al.	2018	Germany	Original Prospective	Orthopaedics	Post	RGW	Activity	O
Physical Activity and Sedentary Behavior in Bariatric Patients Long-Term Post-Surgery	Reid, R. E. R., et al.	2015	Canada	Original Prospective	Bariatric	Post	RGW	Activity, Biometrics	TM
Physical activity levels after limb salvage surgery are not related to clinical scores-objective activity assessment in 22 patients after malignant bone tumor treatment with modular prostheses	Rosenbaum, D., et al.	2008	Germany	Original Prospective	Orthopaedics	Post	RGW	Activity	TM
Multi-segment foot kinematics after total ankle replacement and ankle arthrodesis during relatively long-distance gait	Rouhani, H., et al.	2012	Switzerland	Original Prospective	Orthopaedics	Post	RGW	Biometrics	TM, O
The effect of total knee arthroplasty on joint movement during functional activities and joint range of motion with particular regard to higher flexion users	Rowe, P. J., et al.	2005	UK	Original Prospective	Orthopaedics	Pre, Post	RGW	Biometrics	RP
Energy Harvesting and Sensing with Embedded Piezoelectric Ceramics in Knee Implants	Safaei, M., et al.	2018	USA	Feasibility/Validity	Orthopaedics	Post	RGW	Activity, Biometrics	F
Development and validation of a lower-extremity activity scale. Use for patients treated with revision total knee arthroplasty	Saleh, K. J., et al.	2005	USA	Feasibility/Validity	Orthopaedics	Post	Unknown	Activity	P
Initial ExPerience with Real-Time Continuous Physical Activity Monitoring in Patients Undergoing Spine Surgery	Scheer, J. K., et al.	2017	USA	Original Prospective	Neurosurgery	Post	CGW	Activity	TM
Validation of Activity Tracking Procedures in Elderly Patients after Operative Treatment of Proximal Femur Fractures	Schmal, H., et al.	2018	Denmark	Original Prospective	Orthopaedics	Post	CGW	Activity	O
Quantitative assessment of walking activity after total hip or knee replacement	Schmalzried, T. P., et al.	1998	USA	Original Prospective	Orthopaedics	Post	CGW	Activity	TM
Physical activity after outpatient surgery and enhanced recovery for total knee arthroplasty	Schotanus, M. G. M., et al.	2017	Netherlands	Original Prospective	Orthopaedics	Post	CGW	Activity	TM, O
Step activity monitoring in lumbar stenosis patients undergoing decompressive surgery	Schulte, T. L., et al.	2010	Germany	Original Prospective	Neurosurgery	Pre, Post	RGW	Activity	TM
Horizontal jumping biomechanics among elite male handball players with and without anterior cruciate ligament reconstruction. An inertial sensor unit-based study	Setuain, I., et al.	2019	Spain	Original Prospective	Orthopaedics	Post	RGW	Biometrics	TM
Acceleration and Orientation Jumping Performance Differences Among Elite Professional Male Handball Players with or Without Previous ACL Reconstruction: An Inertial Sensor Unit-Based Study	Setuain, I., et al.	2015	Spain	Original Prospective	Orthopaedics	Post	RGW	Biometrics	TM
Optimal Sampling Frequency for Wearable Sensor Data in Arthroplasty Outcomes RGW. A Prospective Observational Cohort Trial	Shah, R. F., et al.	2019	USA	Original Prospective	Orthopaedics	Post	CGW	Biometrics	P
Step Activity After Surgical Treatment of Ankle Arthritis	Shofer, J. B., et al.	2019	USA	Original Prospective	Orthopaedics	Pre, Post	RGW	Activity	TM
Activity sampling in the assessment of patients with total joint arthroplasty	Silva, M., et al.	2005	USA	Original Prospective	Orthopaedics	Post	CGW	Activity	TM
Dynamic assessment of the wrist after total wrist arthroplasty	Singh, H. P., et al.	2017	UK	Original Prospective	Orthopaedics	Post	RGW	Biometrics	TM
Dynamic assessment of wrist after proximal row carpectomy and 4-corner fusion	Singh, H. P., et al.	2014	UK	Original Prospective	Orthopaedics	Post	RGW	Biometrics	TM
Comparison of the clinical and functional outcomes following 3- and 4-corner fusions	Singh, H. P., et al.	2015	UK	Original Prospective	Orthopaedics	Post	RGW	Biometrics	TM
Quantifying Real-World Upper-Limb Activity Via Patient-Initiated Movement After Nerve Reconstruction for Upper Brachial Plexus Injury	Smith, B. W., et al.	2019	USA	Original Prospective	Neurosurgery	Post	RGW	Activity	F
The effect of electromagnetic navigation in total knee arthroplasty on knee kinematics during functional activities using flexible electrogoniometry	Smith, J. R., et al.	2013	UK	Original Prospective	Orthopaedics	Post	RGW	Biometrics	O
A Randomized Study of Exercise and Fitness Trackers in Obese Patients After Total Knee Arthroplasty	Smith, W. A., et al.	2019	USA	Original Prospective	Orthopaedics	Post	CGW	Activity	O
Objective measurement of function following lumbar spinal stenosis decomPression reveals improved functional capacity with stagnant real-life physical activity	Smuck, M., et al.	2018	USA	Original Prospective	Neurosurgery	Pre, Post	RGW	Activity, Biometrics	TM
Preliminary evidence for physical activity following pelvic exenteration: a pilot longitudinal cohort study	Steffens, D., et al.	2019	Australia	Original Prospective	Surgical Oncology	Post	RGW	Activity	TM
A Cyber-Physical System for Near Real-Time Monitoring of At-Home Orthopedic Rehabilitation and Mobile-Based Provider-Patient Communications to Improve Adherence: Development and Formative Evaluation	Stevens, T., et al.	2020	USA	Feasibility/Validity	Orthopaedics	Post	SP	Activity	F
Reliability of the 6-min walking test Smartphone application	Stienen, M. N., et al.	2019	Switzerland	Feasibility/Validity	Neurosurgery	Post	SP	Activity	F
Wireless Monitoring Program of Patient-Centered Outcomes and Recovery Before and After Major Abdominal Cancer Surgery	Sun, V., et al.	2017	USA	Original Prospective	General Surgery	Pre, Post	CGW	Activity	TM
Clinical Evaluation of Implant-Supported Removable Partial Dentures with a Stress-Breaking Attachment	Suzuki, Y., et al.	2017	Japan	Original Prospective	Oromaxillofacial	Post	CGW	Biometrics	TM, O
A Mobile Health Application to Track Patients After Gastrointestinal Surgery: Results from a Pilot Study	Symer, M. M., et al.	2017	USA	Feasibility/Validity	Colorectal	Post	CGW	Biometrics	TM
Which functional assessments Predict long-term wear after total hip arthroplasty?	Takenaga, R. K., et al.	2013	USA	Original Prospective	Orthopaedics	Post	RGW	Activity	P
Physical Behavior and Function Early After Hip Fracture Surgery in Patients Receiving Comprehensive Geriatric Care or Orthopedic Care-A Randomized Controlled Trial	Taraldsen, K., et al.	2014	Norway	Original Prospective	Orthopaedics	Post	RGW	Biometrics	TM, P
Multiple days of monitoring are needed to obtain a reliable estimate of physical activity in hip-fracture patients	Taraldsen, K., et al.	2014	Norway	Original Prospective	Orthopaedics	Post	RGW	Activity, Biometrics	TM, O
The long-term effect of being treated in a geriatric ward compared to an orthopaedic ward on six measures of free-living physical behavior 4 and 12 months after a hip fracture—a randomised controlled trial	Taraldsen, K., et al.	2014	Norway	Original Prospective	Orthopaedics	Post	RGW	Activity	TM
John Charnley Award: Randomized Clinical Trial of Direct Anterior and MiniPosterior Approach THA: Which Provides Better Functional Recovery?	Taunton, M. J., et al.	2018	USA	Original Prospective	Orthopaedics	Post	RGW	Activity	O
Quantified-Self for Obesity: Physical Activity Behaviour Sensing to Improve Health Outcomes	Taylor, D., et al.	2016	UK	Original Prospective	Bariatric	Pre, Post	SP	Activity	F, TM
The Ambulatory Eye Shield Head Tracking Device with Real-Time Feedback for Gas Filled Eye Patients	Thanawattano, C., et al.	2019	Thailand	Feasibility/Validity	Ophthalmology	Post	SP, RGW	Biometrics	F
Assessment of Physical Activity by Wearable Technology During Rehabilitation After Cardiac Surgery: Explorative Prospective Monocentric Observational Cohort Study	Thijs, I., et al.	2019	Belgium	Original Prospective	Cardiothoracic	Post	CGW	Activity	O
Recovery of mandibular motion after closed and open treatment of unilateral mandibular condylar process fractures	Throckmorton, G. S. and E. Ellis	2000	USA	Original Prospective	Oromaxillofacial	Post	RGW	Biometrics	TM
The monitoring of activity at home after total hip arthroplasty	Toogood, P. A., et al.	2016	USA	Original Prospective	Orthopaedics	Post	CGW	Activity	TM
Normative data of a Smartphone app-based 6-min walking test, test-retest reliability, and content validity with patient-reported outcome measures	Tosic, L., et al.	2020	Switzerland	Feasibility/Validity	Neurosurgery	Post	SP	Activity	O
Evaluation of improvement in quality of life and physical activity after total knee arthroplasty in greek elderly women	Tsonga, T., et al.	2011	Greece	Original Prospective	Orthopaedics	Post	CGW	Activity	TM
Telerehabilitation of Patients with Injuries of the Lower Extremities	Tsvyakh, A. I. and A. J. Hospodarskyy	2017	Ukraine	Feasibility/Validity	Orthopaedics	Post	RGW	Activity	O
Measurement of physical activity in the Pre- and early Post-operative Period after total knee arthroplasty for Osteoarthritis using a Fitbit Flex device	Twiggs, J., et al.	2018	Australia	Original Prospective	Orthopaedics	Pre, Post	CGW	Activity	TM
Measuring physical activity in patients after surgery for a malignant tumour in the leg—The reliability and validity of a continuous ambulatory activity monitor	van Dam, M. S., et al.	2001	Netherlands	Feasibility/Validity	Surgical Oncology	Post	RGW	Activity	TM
Measuring physical activity in patients after surgery for a malignant tumour in the leg. The reliability and validity of a continuous ambulatory activity monitor	van Dam, M. S., et al.	2001	Netherlands	Original Prospective	Orthopaedics	Post	RGW	Activity	TM
Fatigue, level of everyday physical activity and quality of life after liver transplantation	van den Berg-Emons, R., et al.	2006	Netherlands	Original Prospective	Transplant	Post	RGW	Activity	TM
Knee kinematics in functional activities seven years after total knee arthroplasty	van der Linden, M. L., et al.	2006	UK	Original Prospective	Orthopaedics	Post	RGW	Biometrics	TM
Between-day repeatability of knee kinematics during functional tasks recorded using flexible electrogoniometry	van der Linden, M. L., et al.	2008	UK	Original Prospective	Orthopaedics	Post	RGW	Biometrics	TM
Exercise therapy after coronary artery bypass graft surgery: a randomized comparison of a high and low frequency exercise therapy program	van der Peijl, I. D., et al.	2004	Netherlands	Original Prospective	Cardiothoracic	Post	RGW	Activity	RP
Feedback From Activity Trackers Improves Daily Step Count After Knee and Hip Arthroplasty: A Randomized Controlled Trial	Van der Walt, N., et al.	2018	Australia	Original Prospective	Orthopaedics	Pre, Post	CGW	Activity	O
Validation of a novel activity monitor in impaired, slow-walking, crutch-supported patients	van Laarhoven, S. N., et al.	2016	Netherlands	Feasibility/Validity	Orthopaedics	Post	CGW	Activity	F
Individual Patient-reported Activity Levels Before and After Joint Arthroplasty Are Neither Accurate nor Reproducible	Vaughn, N. H., et al.	2019	USA	Original Prospective	Orthopaedics	Post	CGW	Activity	TM
A kinematical analysis of the shoulder after arthroplasty during a hair combing task	Veeger, H. E., et al.	2006	Netherlands	Original Prospective	Orthopaedics	Post	RGW	Biometrics	P
Grammont versus lateralizing reverse shoulder arthroplasty for proximal humerus fracture: functional and radiographic outcomes	Verdano, M. A., et al.	2018	Italy	Original Retrospective	Orthopaedics	Post	RGW	Biometrics	O
Walking and chair rising performed in the daily life situation before and after total hip arthroplasty	Vissers, M. M., et al.	2011	Netherlands	Original Prospective	Orthopaedics	Pre, Post	RGW	Activity	TM
Functional capacity and actual daily activity do not contribute to patient satisfaction after total knee arthroplasty	Vissers, M. M., et al.	2010	Netherlands	Original Prospective	Orthopaedics	Pre, Post	RGW	Activity	O
Function and activity after minimally invasive total hip arthroplasty compared to a healthy population	von Rottkay, E., et al.	2018	Germany	Original Prospective	Orthopaedics	Post	RGW	Activity	TM
Wearable Sensor-Based Digital Biomarker to Estimate Chest Expansion During Sit-to-Stand Transitions—A Practical Tool to Improve Sternal Precautions in Patients Undergoing Median Sternotomy	Wang, C., et al.	2019	USA	Feasibility/Validity	Cardiothoracic	Post	RGW	Biometrics	F
Quantifying the influence of DBS surgery in patients with Parkinson’s disease during Perioperative Period by wearable sensors	Wang, J., et al.	2019	China	Original Prospective	Neurosurgery	Pre, Peri, Post	RGW	Biometrics	TM
Upper extremity function in the free living environment of adults with traumatic brachial plexus injuries	Webber, C. M., et al.	2019	USA	Original Prospective	Orthopaedics	Pre, Post	RGW	Activity, Biometrics	TM
Sedentary Behavior, Cadence, and Physical Activity Outcomes after Knee Arthroplasty	Webber, S. C., et al.	2017	Canada	Original Prospective	Orthopaedics	Post	RGW	Activity	TM, RP
Use of Activity Tracking in Major Visceral Surgerythe Enhanced Perioperative Mobilization Trial: a Randomized Controlled Trial	Wolk, S., et al.	2017	Germany	Original Prospective	General Surgery	Post	CGW	Activity	F
Wearable-Based Mobile Health App in Gastric Cancer Patients for Postoperative Physical Activity Monitoring: Focus Group Study	Wu, J. M., et al.	2019	Taiwan	Feasibility/Validity	Surgical Oncology	Pre, Peri, Post	SP	Activity	F
Assessing function in patients undergoing joint replacement: a study protocol for a cohort study	Wylde, V., et al.	2012	UK	Original Prospective	Orthopaedics	Post	RGW	Activity	TM
Implantable Multi-Modality Probe for Subdural Simultaneous Measurement of Electrophysiology, Hemodynamics, and Temperature Distribution	Yamakawa, T., et al.	2019	Japan	Feasibility/Validity	Neurosurgery	Peri, Post	RGW	Biometrics	F
Sensor-Based Upper-Extremity Frailty Assessment for the Vascular Risk Stratification	Yanquez, F. J., et al.	2020	USA	Feasibility/Validity	Vascular	Post	RGW	Biometrics	RP
Kinematic study of the temporomandibular joint in normal subjects and patients following unilateral temporomandibular joint arthrotomy with metal fossa-eminence partial joint replacement	Yoon, H. J., et al.	2007	South Korea	Original Prospective	Oromaxillofacial	Post	SP, CGW	Biometrics	TM
Biomechanical Gait Variable Estimation Using Wearable Sensors after Unilateral Total Knee Arthroplasty	Youn, I. H., et al.	2018	South Korea	Feasibility/Validity	Orthopaedics	Post	RGW	Biometrics	F
Over-the-top ACL Reconstruction Plus Extra-articular Lateral Tenodesis with Hamstring Tendon Grafts: Prospective Evaluation with 20-Year Minimum Follow-up	Zaffagnini, S., et al.	2017	Italy	Original Prospective	Orthopaedics	Post	RGW	Biometrics	TM
Assessing activity in joint replacement patients	Zahiri, C. A., et al.	1998	USA	Original Prospective	Orthopaedics	Post	Unknown	Activity	TM
Evaluation of Gait Variable Change over Time as Transtibial Amputees Adapt to a New Prosthesis Foot	Zhang, X., et al.	2019	China	Original Prospective	Orthopaedics	Post	RGW	Biometrics	TM

CGW = Consumer-Grade Wearables, RGW = Research-Grade Wearables, SP = Smartphone, F = Feasibility, TM = Tracking or Monitoring, RP = Risk Profiling, O = Optimization, P = Prediction.

## Appendix D

**Table A3:** List of Surgical Specialties and Procedures.

**Bariatric Surgery**	**Obstetrics and Gynecology**	Meniscectomy
Gastric Bypass Surgery	Cesarian Section	Proximal Femur Fracture Fixation
**Breast Surgery**	Hysterectomy	Proximal Row Carpectomy
Mastectomy	**Ophthalmologic Surgery**	Transtibial Amputation
Breast Cancer Surgery	Cataract Surgery	Rotator Cuff Repair
**Cardiothoracic Surgery**	Eye Surgery	Shoulder Surgery
Angioplasty	**Oromaxillofacial Surgery**	Shoulder Arthroplasty
Arterial Catheterization	Dental Implantation Surgery	Shoulder Prostheses Surgery
Cardiac Surgery	Temporomandibular Joint Replacement	Spinal Stenosis Surgery
Coronary Artery Bypass Grafting	Unilateral Mandibular Condylar Fixation	Spine Surgery
Elective Cardiac Surgery	**Orthopedic Surgery**	Total Ankle Arthroplasty
Pulmonary Surgery	3-Corner-Fusion	Total Hip Arthroplasty
Lung Cancer Surgery	4-Corner Fusion	Total Joint Arthroplasty
Lung Lobectomy	Achilles Tendon Rupture Repair	Total Knee Arthroplasty
Lung Resection	ACL Reconstruction Surgery	Total Wrist Arthroplasty
Major Pulmonary Surgery	Ankle Surgery	Vertebroplasty
Sternotomy	Back Surgery	**Surgical Oncology**
Thoracic Surgery	Carpal Tunnel Release	Abdominal Cancer Resection
**Colorectal Surgery**	Decompressive Spine Surgery	Major Oncologic Surgery
**General Surgery**	Endoprosthesis Surgery	Pelvic Exenteration
Abdominal Surgery	Fracture Repair	Sarcoma Resection
Gastric Resection Surgery	Hallux Valgus Correction Surgery	Lower Extremity Tumor Resection
Gastrointestinal Resection	Hip Fracture Surgery	**Transplant Surgery**
Hepatic Resection	Hip Surgery	Elective Organ Transplantation
Hepatobiliary Resection	Knee Prostheses Surgery	Kidney Transplant Surgery
Inguinal Surgery	Limb Salvage Surgery	Liver Transplant Surgery
Major Abdominal Surgery	Lower Extremity Orthopedic Surgery	**Urologic Surgery**
**Neurosurgery**	Lower Limb Amputation Surgery	Cystectomy
Brachial Plexus Nerve Transfer Surgery	Lumbar Decompression Surgery	**Vascular Surgery**
Deep Brain Stimulation	Lumbar Microdiscectomy	Lower Limb Amputation Surgery
Transsphenoidal Surgery	Lumbar Spine Surgery	
Traumatic Brachial Plexus Injury Repair	Malleolar Fracture Fixation	

## Appendix E

**Table A4:** Technologies including Activity Trackers, Smartphone Applications, Research-/Commercial-grade wearables, Other Sensors.

**Research-Grade Wearables and Sensors**	Magnet Sensors (Other)	Activity Tracker/Monitor (Other)	Sportline 345 Pedometer
Actigraph AM7164-2.2 activity monitor	Magnetometer (Other)	Apple Watch	Sportline Pedometer
Actigraph GT1M accelerometer	Micro-Motion Logger System	Axivity AX3	SW200 Yamax Digiwalker Pedometer
ActiGraph GT3X+ Activity Monitors	Microstrain Inertia Link	BioPACK Tracking Device	USB Accelerometer ModelX8M-3
ActiGraph wGT3X-BT accelerometer	MoLab Portable Motion Sensor System	Digi-Walker SW-200 Pedometer	USB accelerometer X16-mini
ActivPAL activity monitor	MTx Inertial Orientation Tracker	Fitbase	Visual Behavior Monitor
ADXL 210 acclerometers	MVN Awinda	Fitbit (Other)	Wavelet Health Wristband
ADXRS 250 gyroscopes	Noraxon accelerometer	Fitbit Charge	Withings Pulse Ox Activity Monitor
AMP-331c Activity Monitor	Nottingham Leg ExtensorPower (LEP)	Fitbit Flex	Yamax FitPro Pedometer
Analog Devices accelerometer	Pedar-X	Fitbit Zip	Yamax SW 200/LS2000 Pedometer
APDM Movement Monitoring System	POHTRACK (Postoperative Head Tracking Device)	Fitness Tracker (Other)	**Smartphone Applications**
Biometrics XM65 Electrogoniometer	RehaGait R System	Garmin (Other)	6WT Application
BioSensics Triaxial Gyroscope Sensors	Saphon Visi-trainer3	Garmin Vivoactive HR device	Beiwe Application
BioStampRC Sensors	SenseWear Pro2	Garmin Vivofit2	Capstesia Application
Dynaport ADL monitor	SenseWear Pro3	GC Dataconcepts LLC Accelerometer Activity Monitor	mHealth Application
Electrogoniometer (Other)	SensiumVitals	HITEC Pedometer	Moves Application by Protogeo
Exfix Accelerometer	Sensors (other)	Lumo Lift Device	POHTRACK (Postoperative Head Tracking Device) Application
Flock of Birds	SG150 Flexible Electrogoniometer	Lumo Run	Rehabilitation Monitoring Application
Footswitches	SHIMMER 2R Sensor Units	MetaWear C Sensor Board	Smartphone accelerometer
GT9X Link ActiGraph	ShoWIder	MiBand2	Spine-Specifc 6WT Application
GWalk Sensor	Sirognathograph by Siemens Corp	MicrosoftKinect v2 sensor	SurgeryDiary Application
Gyroscope (Other)	Sphygmomanometer (Other)	Mio Activity Tracker (Other)	The Motion-Monitor
HipGuard	StepWatch 3™Activity Monitor	Misfit Shine	The NeuroPath Application
IC-3031 Uniaxial Piezo-resistive Accelerometers	Temec Instruments Accelerometer	New Lifestyles NL-800 Pedometer	The RehabTracker Application
Inclination Sensors (Other)	The HealthPatch MD	Omron HJ-321-E Pedometer	TKR Application
Inertial Measurement Unit	The PAM	Omron HJ-720 TE2 Pedometer	WalkOn Application
Intelligent Device for EnergyExpenditure and Activity	The Wake Forest RTLS	Omron Pedometer	**Unknown**
Kenz Lifecoder GS Accelerometer	Vitaport3 accelerometer	Physilog ^®®^ activity monitor	Pedometer (other)
KiRA	**Consumer-Grade Wearables**	Polar Loop activity tracker	
Lifecoder EX Pedometer	3Space Fastrak System	Power Walker EX-510 Yamax Step Counter	
M180 Electrogoniometer	Activ8™ Professional Activity Monitor	Smartwatch (Other)	
